# Bioactive Lichen Secondary Metabolites and Their Presence in Species from Chile

**DOI:** 10.3390/metabo13070805

**Published:** 2023-06-28

**Authors:** Erick Poulsen-Silva, Felipe Gordillo-Fuenzalida, Cristian Atala, Adrián A. Moreno, María Carolina Otero

**Affiliations:** 1 Escuela de Química y Farmacia, Facultad de Medicina, Universidad Andrés Bello, República 252, Santiago 8320000, Chile; e.poulsensilva@uandresbello.edu; 2 Laboratorio de Microbiología Aplicada, Centro de Biotecnología de los Recursos Naturales, Facultad de Ciencias Agrarias y Forestales, Universidad Católica del Maule, Avda. San Miguel 3605, Talca 3466706, Chile; fgordillo@ucm.cl; 3 Instituto de Biología, Facultad de Ciencias, Pontificia Universidad Católica de Valparaíso, Campus Curauma, Av. Universidad 330, Curauma, Valparaíso 2373223, Chile; cristian.atala@pucv.cl; 4 Centro de Biotecnología Vegetal, Facultad de Ciencias de la Vida, Universidad Andrés Bello, Santiago 8370146, Chile; adrian.moreno@unab.cl

**Keywords:** secondary metabolites, biological properties

## Abstract

Lichens are symbiotic organisms composed of at least one fungal and one algal species. They are found in different environments around the world, even in the poles and deserts. Some species can withstand extreme abiotic conditions, including radiation and the vacuum of space. Their chemistry is mainly due to the fungal metabolism and the production of several secondary metabolites with biological activity, which have been isolated due to an increasing interest from the pharmaceutical community. However, beyond the experimental data, little is known about their mechanisms of action and the potential pharmaceutical use of these kinds of molecules, especially the ones isolated from lesser-known species and/or lesser-studied countries. The main objective of this review is to analyze the bibliographical data of the biological activity of secondary metabolites from lichens, identifying the possible mechanisms of action and lichen species from Chile. We carried out a bibliographic revision of different scientific articles in order to collect all necessary information on the biological activity of the metabolites of these lichen species. For this, validated databases were used. We found the most recent reports where in vitro and in vivo studies have demonstrated the biological properties of these metabolites. The biological activity, namely anticancer, antioxidant, and anti-inflammatory activity, of 26 secondary metabolites are described, as well as their reported molecular mechanisms. The most notable metabolites found in this review were usnic acid, atranorin, protolichesterinic acid, and lobaric acid. Usnic acid was the most investigated metabolite, in addition to undergoing toxicological and pharmacological studies, where a hepatotoxicity effect was reported due to uncoupling oxidative phosphorylation. Additionally, no major studies have been made to validate the pharmacological application of these metabolites, and few advancements have been made in their artificial growth in bioreactors. Despite the described biological activities, there is little support to consider these metabolites in pharmaceutical formulations or to evaluate them in clinical trials. Nevertheless, it is important to carry out further studies regarding their possible human health effects. These lichen secondary metabolites present a promising research opportunity to find new pharmaceutical molecules due to their bioactive properties.

## 1. Introduction

Nowadays, the search for new active pharmaceutical molecules from natural sources, such as lichens, has gained considerable attention within the pharmaceutical industry. In order to find new molecules containing new and better biological properties and because of the increasing public demand for sustainable practices, the treatment of new diseases, and the increasing resistance of bacteria and pests to historically effective biomolecules it has been now develop. Lichens can be found growing from tropical regions to the poles, at different elevations, and both in aquatic and dry environments [1]. They grow on different surfaces: over and inside rocks, on soil, and on the surface of trees and animal shells, as well as man-made surfaces, such as concrete, brick, glass, metal, and plastic, among others [2]. All these characteristics are mainly due to their ability to survive extreme temperatures and withstand droughts, floods, salinity, environmental contaminants, and low-nutrient environments [3]. They can even survive exposure to the vacuum of space and cosmic radiation [4] and are likely candidates for exoplanetary colonization. Due to CO_2_ diffusion and algal physiology limitation, these organisms grow very slowly [5] and are difficult to mass-produce under artificial conditions, but recent advancements show promising results in this regard [6].

The symbiotic association of lichens is formed by at least one fungal species, named mycobiont, and a photosynthetic micro-organism, known as photobiont, which is normally a green alga but can also be a cyanobacteria. The mycobiont usually determines lichen morphology and includes species belonging to the Ascomycota and, to a lesser extent, the Basidiomycota. The mycobiont is involved in fixing the lichen to the substrate and mineral and water acquisition. It is also involved in the protection of the photobiont from environmental factors, such as desiccation and exposition to intense light through the production of secondary metabolites. Regarding the photobiont, its main role is the synthesis of organic compounds from carbon dioxide, which are essential for lichen growth [7]. The lichen secondary metabolites have a high grade of diversity, estimated at more than 1000 different compounds [3]. Based on its chemical structure, they can be classified as phenolic compounds, dibenzofurans (i.e., usnic acid), depsides (i.e., atranorin, barbatic acid), depsidones (i.e., salazinic acid, lobaric acid), aliphatic acids (i.e., protolichesterinic acid), quinones (i.e., parietin), pulvinic acid derivatives (i.e., vulpinic acid), and compounds related to anthraquinones, among others [1,8].

Altogether, with the identification of these compounds, there are also several biological activities documented for these secondary metabolites, which include antimicrobial, antioxidant, anticancer, anti-inflammatory, analgesic, antipyretic, antiviral, and photoprotective, among others. Antioxidant activity involves the ability of a compound or enzyme to inhibit the effects of reactive oxygen species (ROS). The process of aging, and different chronic diseases, such as atherosclerosis, Parkinson’s disease, Alzheimer’s disease, and some types of cancer, are related to oxidative damage, and antioxidants act by scavenging ROS, thus preventing damage. Regarding what defines a compound as having “anticancer activity”, it mainly involves a series of cytotoxic, anti-tumor, anti-proliferative, and anti-mutagenic activities, among others. These kinds of biological properties are even more relevant if the cytotoxic effects, for example, are specific toward cancerous cells, leaving non-cancerous cells with no significant effects [3,8].

There are several secondary metabolites that present a single biological activity, such as the antimicrobial effect of vulpinic acid against aerobic and anaerobic micro-organisms; others possess more than one activity, such as in the case of usnic acid, which has antimicrobial, analgesic, and anti-proliferative effects. Usnic acid is, to date, the only metabolite for which the toxicological effects have been extensively studied. However, it has also been found to have toxic effects under certain conditions. Its toxicity appears to be dose-dependent, with higher concentrations leading to more pronounced adverse effects. Despite its potential toxicity, usnic acid has been investigated for its potential pharmaceutical applications, particularly as an antibiotic or anticancer agent [1]. Several of these properties were discovered by experimental studies, including tests against pathogen cultures and in vitro assays on different cell lines to evaluate antioxidant properties and cytotoxicity [3]. However, the vast majority of these studies used samples of lichen extracts, and few studies used pure isolated compounds [9]. Besides, there is scarce information about clinical studies that have evaluated the biological activity of these metabolites, and the most recent studies involve revisions of previous experimental studies, mainly in vitro and in vivo assays [10].

With these properties, lichen secondary metabolites have become a promising source of new pharmaceutical molecules; however, there is still a lack of studies that can validate said proposal beyond determining their biological activities. There are many questions that need clarification, such as those about the mechanism of action of these molecules, if there are differences between a lichen species and the biological activity of their metabolites, or which factors are necessary to consider before thinking about testing their effects in clinical assays. Thus, the goal of the following work is to analyze in detail the biological activities of these secondary metabolites by searching the lichen species that could be involved in their production that are both native or non-native to Chile and analyzing their possible mechanism of action. There are close to 1900 lichen species in Chile [11], but there are chemical studies available for only a few, evidencing a promising research area. Thus, we aim to determine which lichen secondary metabolites could be considered a future candidate for pharmaceutically active molecules based on the results from in vitro and in vivo assays in order to evaluate the possibility of using them in clinical trials.

## 2. Materials and Methods

For this review, the recompiled studies were focused on the secondary metabolites of lichens, their respective species, and their bioactive properties. The publications were obtained from scientific databases such as PubMed, ScienceDirect, and SciELO, using terms “lichen”, “metabolites”, “biological activity”, and “pharmaceutical” as search keywords. In the case that it was required to search for a specific species of lichen, metabolite, or biological activity, they were also used as search terms.

Priority was given to the studies that were published between 2010–2021 as the main reference to determine the properties of the lichens’ secondary metabolites, preferably experimental studies and systematic reviews. Those studies that were published before the established time period will be used as theoretical references about the characteristics of lichens and their compounds. A comparative approach based on publishing date, experimental results, and scientific relevance was used in order to properly analyze all compiled information. Studies exposing biological activities that are not related to possible pharmacological effects (for example, allelopathic, anti-herbivorous, or insecticidal) were excluded.

## 3. Lichen Species of Pharmaceutical Interest

### 3.1. Lichen Species from the Northern Hemisphere of Pharmaceutical Interest

An extensive number of lichen species is documented in scientific studies, which demonstrate an interest in said community, mainly due to their biological activities and their use in ancestral medicine. There are 60 documented genera of lichens that have been traditionally used by different cultures around the world, mainly in North America, Europe, and Asia [12]. For instance, *Usnea* is regarded as the most common lichen genus used traditionally around the world, except in Australia. Other genera include *Cladonia*, *Ramalina*, *Lobaria*, and *Pertigera*, which are used in North America, Europe, and Asia. The genus *Evernia* and *Pseudevernia* have been traditionally used in Europe and North Africa; meanwhile, *Umbilicaria* is the most common in North America and Asia. *Xanthoparmelia* is another genus that has been used in North America and also in Africa. Some other genera of lichens, such as *Letharia*, *Lethariella*, *Cetraria*, *Parmotrema*, and *Thamnolia*, have been described in specific regions of North America, Europe, and Asian countries, such as China and India. All of these lichens mentioned above have been employed in the treatment of external injuries as disinfectant (antibiotic) and for antihemorrhagic purposes or to treat skin afflictions, such as skin infections or sores. Some species are prepared as a decoction to drink with the purpose of alleviating digestive or respiratory ailments. Other traditional uses that are also associated with these lichen genera involve the treatment of ocular, foot, and obstetric problems (related to complications during birth or contraceptive purposes).

From a taxonomic point of view, the Parmeliaceae family is recognized to represent the great majority of lichen species from a cultural and scientific point of view. These include around 2700 species (equivalent to 10% of the total of the lichen species), which are distributed in close to 80 genera. Among these are *Cetraria*, *Cetrariella*, *Evernia*, *Hypogymnia*, *Lethariella*, *Parmelia*, *Parmotrema*, *Pseudevernia*, *Usnea,* and *Xanthoparmelia* as the most investigated species. This lichen family is well-known for its global distribution and for presenting a diverse number of secondary metabolites, which are used for chemotaxonomic purposes. At a phylogenetic level, seven clades are recognized in the Parmeliaceae family: alectorioid, cetrarioid, letharioid, hypogymnioid, parmelioid, psiloparmelioid, and usneoid [13,14]. Xu et al., 2016, described that the cetrarioid clade is one of the most studied within the Parmeliaceae family, focusing their review on their pharmaceutical potential [15]. In said work, they also mentioned other genera, such *Flavocetraria*, *Masonhalea*, *Nephromopsis,* and *Vulpicida*, as well as cetrarioid lichens that have also been used in traditional medicine. Nonetheless, research on medicinal lichens is not restrained to this particular family, and species belonging to other families have also been studied. *Ramalina* is a genus of cosmopolitan lichens that is composed of more than 240 studied species, mainly because they were used in ancestral medicine to treat injuries, bleeding, inflammations, and asthma [16]. *Cladonia* has also been used traditionally to treat colds, arthritis, fevers, jaundice, constipation, and coughs, among other discomforts [17]. The *Lobaria* genus has been used mainly to treat lung ailments, and *Umbilicaria* has been used to treat stomach aches and external and internal bleeding [12].

One of the most relevant species is *Cetraria islandica*. This lichen, also known as “Icelandic moss”, has been included in European pharmacopoeias since 1500 and has been used traditionally to treat lung diseases, cold symptoms, and gastroenteritis; it also has been used as an antitussive, antituberculosis, and an oropharyngeal anti-inflammatory and for the treatment of skin injuries. In Iceland, it is prepared as an herbal tea or in decoction with milk; it is also dried and prepared as a powder to be used as capsules. It possesses a bitter flavor that can be eliminated with alkalis or by heating in water [12]. Grujičić et al., 2014, performed an in vitro evaluation of the methanol extract of this species, and it was found that *C. islandica* possess slight antimicrobial, antioxidant, and also cytotoxic activity against the two cancer cell lines FemX and LS174. According to the authors, the compounds present in this lichen that are responsible for these activities are lichesterinic acid, protolichesterinic acid, f*umarprotocetraric* acid, lichenine, isolichenine, ascorbic acid, and vitamins B1 and B12, among others [18]. 

Another remarkable lichen is *Pseudevernia furfuracea*, used in ancient Egypt in the process of embalming mummies for aromatic purposes. For this reason, this lichen has been used since 1900 in the perfume industry. In the province of Kütahya, Turkey, this lichen is mixed with clay in order to prepare a paste for topic injury treatment. Experimental assays have determined that *P. furfuracea* possess antioxidant, anti-inflammatory, and wound-healing properties [19]. Another relevant lichen is *Usnea longissima*, a species that has been used in traditional medicine in India for its analgesic, cardiotonic, digestive, and wound-healing properties. Several of these biological properties are associated with the presence of depsides and dibenzofuran compounds, mainly usnic acid. Other prominent metabolites present in this species are barbatic acid, diffractaic acid, and salazinic acid [20]. *Lobaria pulmonaria* is a lichen that has been used to treat lung aches since 1400 in Europe; however, there are also some reports of its use in Turkey, India, and China. Altogether, with its use to treat coughs, asthma, and tuberculosis, it also has been used as a laxative, antiseptic, antihemorrhagic, and digestive compound [12].

### 3.2. Chilean Lichen Species of Pharmaceutical Interest

In the case of Chile, the concept of lichens being used for medicinal purposes is not entirely unknown. The Mapuche and Tehuelche people, indigenous people of Chile, used the “barba de la piedra” or “barbas de viejo” (*Usnea* and *Protousnea* spp.) in order to treat cough symptoms. This lichen was also employed for gastrointestinal, respiratory, cardiovascular, urinary, and obstetric problems. Furthermore, the Selk’nam people used the species *Cladina laevigata* and *Protousnea magellanica* for personal hygiene to wash and dry the body, respectively [12,21].

Different authors have published reports about the species present in Chilean territory and the Antarctic Continent, specifically the Antarctic Peninsula. One of the most highlighted was a series of publications carried out by a team of researchers from Universidad de Valparaíso and Universidad Técnica Federico Santa María around 1980. In these works, a large variety of species were studied, and chemical characterization was performed, as well as the isolation of secondary metabolites by using a series of organic solvents and by means of different techniques, such as thin-layer chromatography and column chromatography. In Table 1, a brief summary of the metabolites found in these species is shown, mentioning only those compounds of pharmaceutical interest that were found in high quantities, which will be addressed in detail later on [22,23,24,25,26,27,28,29,30,31]. In one of these reports, Quilhot et al., 1989, determined the main secondary metabolites of 17 lichen species collected from the South Shetland Islands, where the metabolites usnic acid and atranorin were found in most of the studied species [30]. Two years later, an advance on this research was published, adding 11 other species of Antarctic lichens, finding more secondary metabolites in the species included in the previous study [31]. In 1993, Garbarino revised these 28 species again, declaring the predominance of usnic acid, atranorin, and also the metabolite peroxiergosterol; however, a clear chemical heterogeneity exists in these species, finding compounds such as depsides, depsidones, and dibenzofurans in different concentrations, ranging from traces to a 1% of the lichen’s dry weight [25]. As mentioned earlier, close to 1900 species of lichens are present in Chile [11], evidencing a large gap in the study of their metabolites and chemical composition, likely due to a lack of specialists (lichen taxonomist, systematics mainly) in the country.

Years later, several of these species and their metabolites were evaluated in different experimental studies. Brisdelli et al., 2012, investigated the cytotoxic and in vitro antioxidant activity of six metabolites present in continental and Antarctic Chilean species: diffractaic acid (*Protousnea magellanica*), vicanicin (*Psoroma pallidum*), lobaric acid (*Stereocaulon alpinum*), variolaric acid (*Ochrolechia deceptionis*), protolichesterinic acid (*C. aculeata*), and usnic acid (*Cladonia lepidophora*). Using cell lines derived from colon, cervical, and breast cancer, increasing concentrations of the compounds were administered for 48 H. Then, it was determined that usnic and protolichesterinic acid exerted a strong cytotoxic effect in the three cell lines, significantly decreasing cell survival. The other compounds only exerted moderate effects on a determined group of cells; meanwhile, variolaric acid did not affect the cell viability of the test models. Regarding the results of the antioxidants assay against the 1,1-diphenyl-2-picrylhydrazyl (DPPH) radicals, none of the metabolites exhibited significant activity of radical scavenging [32]. Another study tested the anticancer potential of the metabolites atranorin, gyrophoric, and physodic acid in models of cell lines of human melanoma. It was revealed that the atranorin and gyrophoric acid showed slow inhibitory activity at high concentrations (20–50 μM); meanwhile, physodic acid (isolated from *Hypogymnia lugubris*) showed inhibitory, dose-dependent activity in these cellular models [33].

Other native species and their compounds have been analyzed for their biological activities. For instance, a metabolite with potential anticancerogenic activity is physciosporin, isolated from *Pseudocyphellaria coriacea*, found in the south of Chile. In an in vitro assay using human lung cancer cell lines, this compound presented inhibitory activity against cell migration, showing antimetastatic potential. Another in vitro assay, this time with cancer stem cell lines, showed that the crude extract of the lichen and physciosporin were able to inhibit colony formation, the ability for proliferation, and self-renewal properties [34,35]. Lastly, Yang et al., 2018, performed studies on tumidulin isolated from *Niebla* sp., recollected from the south of Chile. In this study, the anticancer potential of the crude extract and the isolated compound was evaluated against cancer stem cell lines, revealing that this compound also reduced their self-renewal potential [36]. 

### 3.3. Lichen Secondary Metabolites

**Figure 1:**
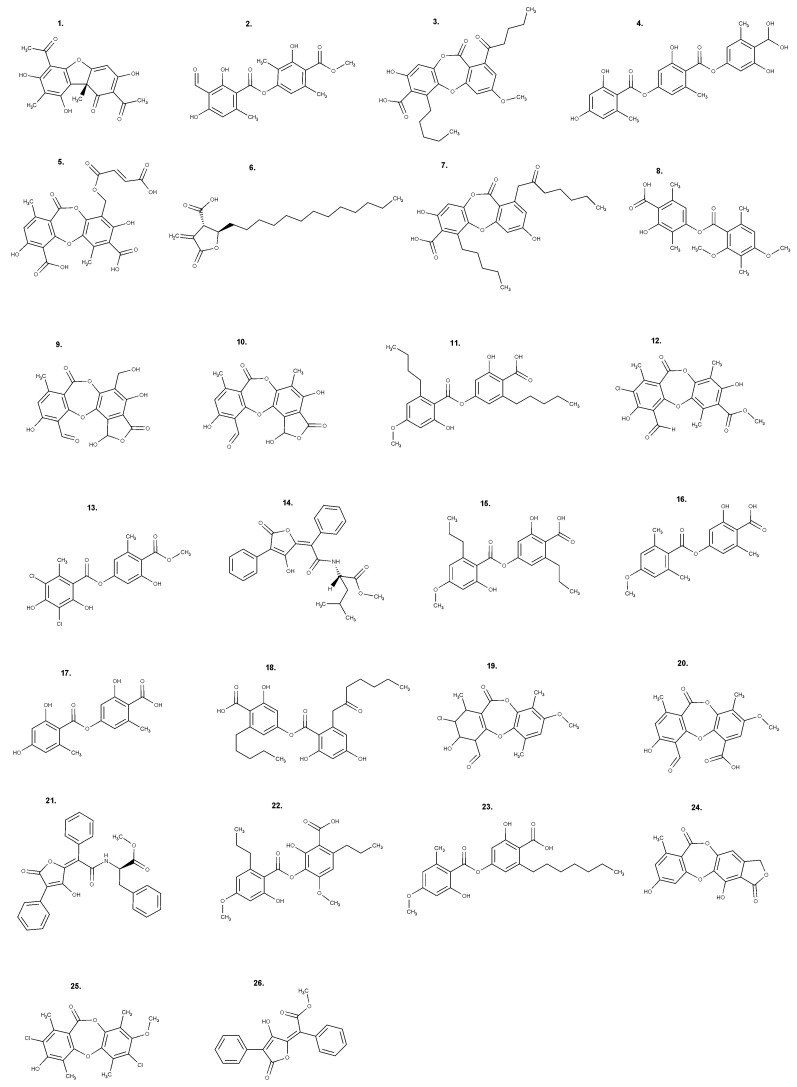
Chemical structures of lichen secondary metabolites described in this review. 1: usnic acid; 2: atranorin; 3: lobaric acid; 4: gyrophoric acid; 5: fumarprotocetraric acid; 6: protolichesterinic acid; 7: physodic acid; 8: diffractaic acid; 9: salazinic acid; 10: norstictic acid; 11: perlatolic acid; 12: physciosporin; 13: tumidulin; 14: epanorin; 15: divaricatic acid; 16: evernic acid; 17: lecanoric acid; 18: olivetoric acid; 19: pannarin; 20: psoromic acid; 21: rhizocarpic acid; 22: sekikaic acid; 23: sphaerophorin; 24: variolaric acid; 25: vicanicin; 26: vulpinic acid.

Most of the secondary metabolites produced by lichens originate from three major biosynthetic pathways: the acetyl-malonate (also known as polyketide pathway), mevalonate, and shikimate pathways. The pathway of acetyl-malonate is the one that produces the most studied compounds in terms of their biological activities, such as depsides, depsidones, and dibenzofurans [7]. Figure 1 shows the chemical structure of the relevant secondary metabolites described in this review, for which the biological properties are reported as follows.

### 3.4. Usnic Acid

Usnic acid is one of the most studied secondary metabolites, isolated for the first time in 1884. It is a dibenzofuran derivative, a cortical pigment of yellow color. It is widely distributed in several families of lichens and is found in great amounts in some of these species. This metabolite possesses two enantiomeric forms which differ slightly in their biological activities: usnic acid (+) and usnic acid (-) [37]. Besides, it is one of the few lichen secondary metabolites that is commercially available from chemical suppliers.

**Figure 2:**
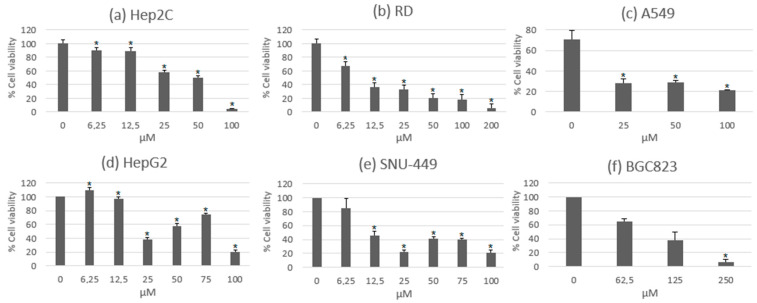
Effect of different concentrations of usnic acid in cell viability of cancer cell lines. In these graphics, the percentage of cell viability in cervical (**a**), rhabdomyosarcoma (**b**), lung (**c**), hepatocellular (**d**,**e**), and gastric (**f**) cancer cell lines are shown. * *p* < 0.05. Data modified from [38,39,40,41].

One of the most studied characteristics of usnic acid is its anti-proliferative activity. Solárová et al., 2020, extensively collected the potential anticancer activities of usnic acid and other metabolites based on several experimental assays using different cell lines [10]. In some of these assays, usnic acid exerted a significant reduction in cell viability on hepatocellular, rhabdomyosarcoma, cervical, lung, and gastric cancer cell lines at different concentrations. These data are shown in Figure 2, where high concentrations of usnic acid exert a significant cytotoxic effect [38,39,40]. One study performed a comparison between the usnic acid isolated from *Usnea diffracta* and a commercial form of it. In these results, both of the metabolite samples showed activity over similar concentration ranges [38]. This is relevant since many of the previous experimental assays were carried out with commercial usnic acid. Alongside the reduction in cell viability, the compound was also able to diminish the growth and cell proliferation of lung and gastric cancer cell lines [39,40,41].

In most of the studies on carcinogenic cell lines, the development of apoptosis after treatment was observed. Apoptosis is a process of programmed cell death by means of a cascade of molecular events. Usnic acid was able to have an effect on the cell lines of ovarian [9], hepatic [38], gastric [40], and breast cancer [42], modulating the expression of the genes that encode proteins, such as Bcl-2 (antiapoptotic), p53 (tumor suppressor gene), Bax (proapoptotic), and caspase-3 (its activation produces the process of apoptosis), among others. The development of apoptosis can also be triggered by means of mitochondrial membrane potential (MMP) depolarization. Usnic acid provoked MMP depolarization in lung, breast, ovarian, and colon cancer cell lines [9,41,42]. Another mechanism that produces apoptosis in cancer cells is intracellular ROS increase. In assays with colon and breast cancer cell lines, usnic acid significantly increased ROS levels in a dose-dependent manner [7,42].

**Figure 3:**
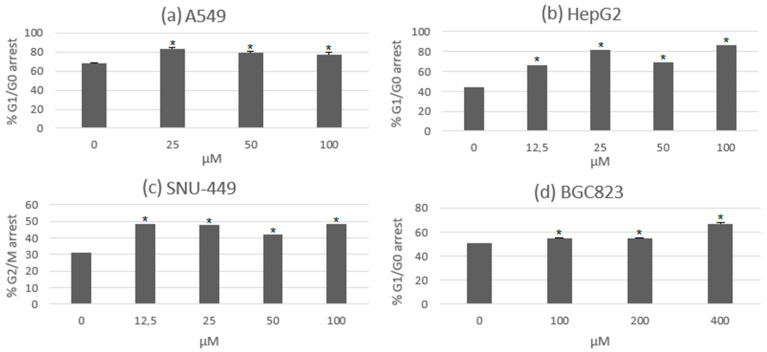
Effect of usnic acid at different concentrations on cell cycle arrest of cancer cell lines. In these graphics, the lung (**a**), hepatic (**b**), and gastric (**d**) cancer cell lines suffered a large amount of cell cycle arrest in the G0/G1 phase, while the hepatocellular cancer cell line SNU-449 (**c**) suffered arrest in the G2/M phase. * *p* <0.05. Data modified from [39,40,41].

Cell cycle arrest in certain phases has been one of the mechanisms of growth and cell proliferation inhibition in several anticancer agents. The progression of the cell cycle is measured by cyclins and cyclin-dependent kinases (CDK), and the modulation of these factors is related to the mechanism of cell cycle arrest. These activities were observed in experimental assays of usnic acid with lung, gastric, and hepatic cancer cell lines. The most significant action of these results was the arrest of the cell cycle in the G0/G1 phase, which was observed in most of the cancer cells. Interestingly, there was an arrest in the G2/M phase in the hepatocellular cancer cell line SNU-449. This might be due to differences in the cell genome and the altered expression of genes related to the cell cycle. In Figure 3, the percentage of cells that suffered cell cycle arrest under the treatment of usnic acid during both the G0/G1 and G2/M phases can be seen [39,40,41].

All the previous work was performed using in vitro assays; however, two of them were also conducted using in vivo assays in order to evaluate the antitumor activity of usnic acid. Geng et al., 2018, performed tests using BALB/c nude mice, which were inoculated with gastric cancer cell lines subcutaneously. One group of mice was treated with usnic acid and the other with 5-fluorouracil, a drug commonly used in chemotherapy, once every 2 days for 11 days. During the treatment process, body weight and tumor volume were measured. In the group treated with usnic acid, there was a significant decrease in the size and weight of the tumor. However, there was no loss of body weight, unlike the group treated with 5-fluorouracil, which suggests that the metabolite performs its antitumoral activity without causing toxicity in the animal model [40]. A similar assay was carried out by Zuo et al., 2014, where breast cancer cells were employed. Similar to the above-mentioned work, a significant reduction in tumor growth after the treatment with usnic acid was observed, associated with apoptotic activity induced by oxidative stress. In this assay, this was compared with the antitumor effect of cyclophosphamide, which also provoked a loss in body weight in the animal model. On the other hand, this phenomenon was not observed in the treatment using usnic acid [42].

Another characteristic of usnic acid is its potential as an antioxidant. In the work of Behera et al., 2012, it was found that, in DPPH assays, the metabolite at a concentration of 0.2 mg/mL had a free radical scavenging activity (51.21%) that was concentration dependent, as well as its nitric oxide (53.19%) and lipid peroxidation (46.57%) scavenging activity, evaluated with sodium nitroprusside and 2,20-azo-bis(2-amidinopropane) dihydrochloride (AAPH) assays, respectively [43]. In a more recent experimental study, the radical inhibitory capacity was evaluated by means of assays with DPPH, 2,2’-azino-bis(3-ethylbenzothiazoline-6-sulfonic) acid (ABTS^+^), superoxide anion (O_2_^-^), and *N,N*-dimethyl-p-phenylenediamine (DMPD^+^), and the results showed significant inhibition was found with a mean inhibitory concentration (IC_50_) of 49.50 µg/mL for DPPH, 10.41 µg/mL for ABTS^+^, 20.38 µg/mL for O_2_^-^, and 33.0 µg/mL for DMPD^+^ [44]. Fernández-Moriano et al., 2017, decided to evaluate the antioxidant capacity of usnic acid in cell lines derived from the central nervous system (SH-SY5Y and U373-MG) when treated with hydrogen peroxide after determining the optimal concentrations of the metabolite that did not cause cytotoxicity [45]. After pretreatment for 24 H, where cell viability was reduced to 55–60% when treated with hydrogen peroxide, it was found that usnic acid was able to reverse these effects, recovering the cell viability of U373-MG by 9.33% at 2.5 µg/mL, and SH-SY5Y by 13.39% at 1 µg/mL of usnic acid. This same trial analyzed the mechanisms of its antioxidant activity and found that usnic acid was capable of moderately increasing the levels of the Nrf2 protein in both cell lines. This protein is part of a metabolic pathway that activates the expression of antioxidant enzymes, such as superoxide dismutase, which was also found to increase under treatment with usnic acid.

Regarding the redox nature of the metabolite, Rabelo et al., 2012, studied TRAP, free radical scavenging capacity, and its activity in SH-SY5Y cell lines under treatment with hydrogen peroxide. In the results, significant antioxidant activity was observed at 20 µg/mL of the metabolite, as well as the effective scavenging activity of hydroxyl radicals and nitric oxide. However, in the in vitro assay using SH-SY5Y cells, when the cells were treated at this concentration, there was a cessation of neuritic processes after 1 H and a loss of cell viability after 4 H. In the test, in conjunction with hydrogen peroxide, cell viability had already decreased after 1 H. The researchers wanted to determine if usnic acid induced the appearance of ROS at the intracellular level, and indeed, this was the case. The author concludes that usnic acid has dual behavior, acting both as an antioxidant and as a prooxidant, according to cellular conditions [46].

In relation to its antioxidant properties, usnic acid also has some photoprotective capacities. In an in vitro assay based on spectrophotometric data, the photoprotection index of usnic acid isolated from the *Vulpicida pinastri* species was determined, and the metabolite was found to be able to act as an ultraviolet radiation filter, specifically UV-B. In addition to this, it was also shown that it was photostable under UV radiation, and that it did not cause phototoxicity or cytotoxicity in HaCaT keratinocyte cell lines [47].

There are also studies that state that usnic acid has anti-inflammatory activity. Among the most recent, a study carried out using in vivo tests to search the effect of usnic acid on acute lung damage induced by lipopolysaccharide (LPS) carried out in Kunming mice as animal models. LPS is a component of the outer membrane of Gram-negative bacteria that induces inflammatory responses, and in the results of pretreatment with usnic acid, it was found that mortality in the animal model was significantly reduced with a dose of 50–100 mg/Kg, in addition to reducing LPS-induced pulmonary edema, decreasing the levels of macrophage and neutrophil cells, and markedly reducing the expression of tumor necrosis factor alpha (TNF-α) and inflammatory cytokines such as IL-6 and IL-8 [48]. Another study performed in vitro tests using RAW264.7 cell line also treated with LPS. The results of this study were similar to the previous one in terms of the significant reduction in the secretion of pro-inflammatory proteins (TNF-α, IL-1β, and IL-6), but usnic acid was also able to strongly reduce cyclooxygenase-2 (COX-2) levels, an enzyme involved in the inflammation process; and also, significantly increase the levels of the anti-inflammatory mediator HO-1. Usnic acid was found to act by blocking the activation of NF-κB, a nuclear transcription factor that regulates the expression of proinflammatory genes, suggesting that its anti-inflammatory effect is due to this action [49].

In relation to its anti-inflammatory activity, the analgesic activity of usnic acid was evaluated by Sujatha et al., 2020, through in vivo tests with Swiss mice for the induction of nociception by injection of capsaicin, glutamate, and formalin in the dorsal area of the right paw. When usnic acid was orally administrated, it exhibited a dose-dependent antinociceptive effect against the three compounds used, evidenced by a decrease in nociceptive behavior in the animal model. This same study found, by means of in silico coupling assays, that usnic acid possessed a comparable degree of binding with anti-inflammatory drugs on COX, nitric oxide synthase (iNOS), and other inflammatory enzymes [50]. It also confers wound-healing potential, where, in an in vivo test with Wistar mice, the sodium form of usnic acid was used. In these results, the metabolite significantly increased the degree of healing in circular incisions made on the back of the animal models. Histological analysis showed that it caused less infiltration of inflammatory cells and increased proliferation of fibroblasts, granulation tissue formation, and vascular regeneration, which led to early re-epithelialization of scar tissue. The metabolite was found to cause an increase in vascular endothelial growth factor (VEGF) from the first day of post-incision treatment, reaching a peak on the third day of treatment [51].

This metabolite has also been shown to have inhibitory activity against some relevant enzymes. In one study, it was able to non-competitively inhibit the β-hydroxy-β-methylglutaryl coenzyme A reductase (HMGR) enzyme, which is associated with cholesterol synthesis. Similarly, usnic acid inhibited the angiotensin-converting enzyme (ACE) in the same study, which is the target of a large family of antihypertensive drugs. Based on these results, a certain cardioprotective potential is associated with usnic acid [43]. In another experimental study, usnic acid was shown to have inhibitory activity against the acetylcholinesterase (AChE) enzyme, which is the target for the treatment of Alzheimer’s disease. Its action was compared with the standard inhibitor tacrine, thus demonstrating an effective inhibitory profile against AChE [45]. In the test of Verma et al., 2012, the methanol extract of *Ramalina celastri* was able to inhibit the enzyme α-glucosidase by 69.25%. This enzyme is involved in the digestion of carbohydrates in the small intestine, transforming them into glucose that is incorporated into the blood, and for which inhibition suppresses postprandial hyperglycemia, being the target of some antidiabetic drugs such as acarbose. The extract contained usnic acid, and based on kinetic studies performed in the paper, the metabolite acted as a non-competitive inhibitor of the enzyme [52].

### 3.5. Atranorin

Atranorin is a depside derivative of beta-orcinol, which is also present in several families of lichens, although it has also been found on rare occasions in mosses and higher plants. This was first isolated in 1898, and since then, its biological activities and pharmacological potential have been studied [53].

The anticancer potential of atranorin was evaluated in various in vitro assays. Bačkorová et al., 2011, tested nine cancer cell lines and found that, at a concentration of 50 µM, it was able to exert cytotoxicity only on promyelocytic leukemia cells, but after increasing this concentration, it managed to affect seven other cancer cell lines, such as breast, colon, ovarian cancer, and lymphoid leukemia [54]. In another study by the same author, it was shown that atranorin was capable of reducing MMP, causing a loss of cell membrane integrity and modulating the expression of genes of proteins associated with apoptosis. However, this activity was more effective in ovarian cancer cell lines, whereas for colon cancer cell lines, higher concentrations were needed for a more significant effect [9]. In an assay with hepatocellular cancer cell lines, it was also found that cell growth inhibition was only significant at higher doses of atranorin, also causing cell cycle arrest with a greater proportion in the G2/M phase and inducing cell death mediated by lysis. Furthermore, the metabolite was found to be able to suppress the oncogenic potential of these cell lines by suppressing cell migration and invasion [55]. Atranorin was also able to inhibit cell proliferation and migration, as well as modulate the architecture of the actin cytoskeleton in prostate cancer and melanoma cell lines, thus affecting cell motility [56].

Zhou et al., 2017, performed in vitro and in vivo assays with three lichen species against lung cancer cell lines [57]. The only species that achieved inhibitory activity was *Everniastrum vexans*, where atranorin was isolated as the main component of its acetone extract. It was confirmed that the activity of the metabolite coincided with that of the crude extract and this was able to inhibit cell migration and invasion by 46% and 33%, respectively, at a dose of 5 µg/mL. The metabolite was capable of inhibiting the Wnt signaling pathway, which is associated with cell motor activity and the expression of genes that regulate cell cycle and metastatic activity. It also dose-dependently inhibited AP-1 activation mediated by KITENIN, a protein that promotes tumor progression and invasive potential, and also inhibited various factors associated with cell proliferation, survival, and motility, such as Rho-GTPase and STAT. Using in vivo tests with mouse models xenotransplanted with Lewis lung carcinoma cell lines, a reduction in tumor volume and weight was observed, as well as a reduction in the genes mentioned above.

Atranorin has also been evaluated for its antioxidant properties. Melo et al., 2011, did several free radical inhibition tests, including hydroxyl radicals, hydrogen peroxide, superoxide, and nitric oxide, along with total reactive antioxidant potential (TRAP) assays, total antioxidant reactivity (TAR) index, and in vitro lipid peroxidation assays. In the TRAP and TAR trials, atranorin was found to have a dose-dependent antioxidant capacity, with significant results at doses of 100 µg/mL. As for the free radical scavenging results, there was significant degradation regarding superoxide radical formation. However, atranorin significantly increased hydrogen peroxide formation in vitro and enhanced lipid peroxidation induced by the free radical-producing compound AAPH. In addition, no inhibitory effects were observed against hydroxyl radicals and nitric oxide; moreover, there was an increase in the formation of nitrites with increasing doses of atranorin. These results show that atranorin has dual behavior between antioxidant and pro-oxidant, and to corroborate, once again, its antioxidant potential, an assay was carried out with cell lines derived from the central nervous system induced by oxidative stress. When treated with 400 µM hydrogen peroxide for 3 H, there was a 40% loss in viability in the control group, but when they were treated together at different doses of atranorin, it was observed that the metabolite protected, in a dose-dependent manner, the cytotoxic effects of hydrogen peroxide. Furthermore, by itself, atranorin did not exert cytotoxicity on cell lines. The author states that this differential activity may depend on the composition of the system in which they were found and that further analysis is needed to understand the redox properties of these compounds that exert this dual behavior [58]. Regarding the photoprotective properties of atranorin, Lohézic-Le Dévéhat et al., 2013, performed in vitro tests on this and other metabolites and found that atranorin had a weak photoprotective capacity when compared to the rest of the secondary metabolites of lichens. This is based on the results of sun protection factor and UVA protection factor [59].

The anti-inflammatory activity of atranorin has been evaluated in both in vitro and in vivo studies. Its potential was evaluated by Melo et al., 2011, in an in vivo study with carrageenan-induced paw edema assays in mice, and it was found that the metabolite was able to significantly reduce the edema volume to 29.3–32.9% at doses of 100–200 mg/Kg, respectively. In this same study, a leukocyte migration assay induced by carrageenan injection into the peritoneal cavity of the animal model was performed. After microscopic examination, it was found that all doses of atranorin had a significant effect in reducing the number of leukocytes, presenting a percentage of inhibition of 31.9, 35.9, and 42.5% at doses of 50, 100, and 200 mg/Kg, respectively. The authors of this study suggest that atranorin was capable of inhibiting the synthesis of the different inflammatory mediators involved in cell migration, supporting their proposal in previous studies that showed that the metabolite could inhibit COX enzymes and also inhibit the biosynthesis of leukotriene B4. However, they claim that more studies are needed to determine the mechanism of action of this anti-inflammatory activity [60].

Siqueira et al., 2010, evaluated the antinociceptive capacity of atranorin in vivo in oropharyngeal pain induced by formalin and capsaicin, using Swiss mice as the animal model. Pretreatment with different doses of atranorin achieved pronounced antinociception, evidenced by a decrease in nociceptive behavior in the animal. The dose that achieved the greatest antinociceptive effects, 400 mg/Kg, was evaluated again in an in vivo trial, together with the antagonist naloxone, in order to confirm if there was activity in the morphinomimetic receptors. It was observed that the effect of atranorin was inhibited by naloxone, showing greater nociceptive results in animal models, suggesting that atranorin has a central analgesic effect [61]. Another study evaluated the wound healing activity of atranorin in vivo, performing excision wounds in Wistar mice that were treated with an ointment containing the metabolite at concentrations of 1% and 5%. In their results, they found that the topical administration of atranorin significantly decreased the area of the wound in a dose-dependent manner, in addition to accelerating the healing process, increasing the formation of granular tissue and collagen, and increasing the formation of fibroblasts and capillaries. The metabolite was also able to modulate the inflammatory profile of the wound, reducing the number of neutrophils when compared to the control group [62].

### 3.6. Lobaric Acid

Lobaric acid is an orcinol-type depsidone that has been isolated from different Antarctic lichens, mainly the native species *Stereocaulon alpinum* [1,63]. Many of the works mentioned below carried out tests with this lichen species.

The anticancer potential of lobaric acid has been evaluated in studies with cell lines of cervical and colon cancer, achieving significant cytotoxicity at high concentrations [32,64]. In one of the previous studies, the team of Hong et al., 2018, observed, by light microscopy, a decrease in the number of cells and an alteration in cell morphology. The cancer cells had an elongated shape and filamentary protuberances, typical signs of apoptosis, according to the authors of the work. Finally, apoptotic activity was confirmed by means of an annexin V staining assay and by the discovery of its ability to modulate apoptosis-mediating proteins (Bcl-2 and PARP). Along with that, it was found that the metabolite also caused cell cycle arrest in the G2/M phase [64]. Emsen et al., 2018, carried out tests with glioblastoma multiforme cell lines and the activity of lobaric acid in these cells, testing the release of lactate dehydrogenase (LDH), which is an enzyme that is released into the medium as a result of damage, occurring during apoptosis. In their results, lobaric acid achieved an increase in LDH production in glioblastoma cell lines and obtained an IC_50_ value of 5.77 mg/L. However, this metabolite also caused cytotoxic effects in primary rat cerebral cortex cell lines, which were used as a normal cell model (IC_50_ of 9.08 mg/L). Based on the total oxidative status assay, lobaric acid caused a high pro-oxidative activity in these cell lines [65].

Lobaric acid was found to possess superoxide radical scavenging activity, comparable to positive controls of hydroxybutylanisole (BHA) and propyl gallate compounds [66]. The immunomodulatory and enzymatic inhibitory capacity of lobaric acid was also evaluated, and it was found that the metabolite has a moderate capacity to inhibit AChE (IC_50_ of 26.86 µM) and significant inhibition of the phosphodiesterase enzyme was also demonstrated (IC_50_ of 313.7 µM). Phosphodiesterase is believed to be involved in biological and pathological processes, such as bone formation, insulin resistance, and cancer cell metastasis, for which the inhibitors are used in the treatment of some types of arthritis. Lobaric acid was also able to inhibit myeloperoxidase-dependent and -independent ROS production, based on a luminol-enhanced chemiluminescence assay in neutrophils and polymorphonuclear leukocytes [67].

The anti-inflammatory activity of lobaric acid was evaluated using assays with LPS-stimulated macrophages. Among the results of one of these studies, it was found that lobaric acid was able to inhibit the activation of the NF-κB signaling pathway in a concentration-dependent manner, with complete inhibition at 50 µM. Strong and significant inhibition of TNF-α secretion was also induced at a concentration of 20 µM. The presence of a carbonyl group on one of the aromatic rings is presumed to provide additional stability in the interaction with NF-κB binding sites. In order to further evaluate the anti-inflammatory potential of lobaric acid, its ability to inhibit the secretion of cytokines was tested, and it was found that the metabolite was able to strongly suppress the secretion of IL-6, IL-8, and IL-1β at concentrations between 20–80 µM. Lobaric acid was also found to act as an agonist for peroxisomal proliferator-activated receptor-gamma (PPAR-ɣ), for which agonists are associated with the reduced expression of pro-inflammatory cytokines and the inhibition of NF-κB activation [68]. Lee et al., 2019, carried out similar tests, where it was also found that the metabolite suppresses prostaglandin E2, the enzyme COX-2, and the production of nitric oxide induced by LPS, inhibiting the expression of iNOS. Lobaric acid was also able to inhibit the expression of the inflammasome protein NLRP3, the formation of which is associated with the maturation and release of some cytokines. NF-κB activation is associated with the mitogen-activated protein kinase (MAPK) signaling pathway, playing a role in the process of inflammation. For this reason, they evaluated whether lobaric acid plays a role in the MAPK pathway, and it was found that the metabolite suppresses the phosphorylation of the mediators of this pathway, such as ERK1/2, JNK1/2, and p38. After evaluating, by means of MAPK inhibitors, if this contributes to the activation of NLRP3, it was found that the signaling pathway is important in the activation of NLRP3 induced by LPS and that lobaric acid suppresses both its activation and the production of pro-inflammatory mediators via MAPK inhibition [69].

Lobaric acid was also one of the seventeen natural compounds of lichens that demonstrated inhibitory activity against the formation of advanced glycation end products (AGEs), the formation of which is correlated with the presence of diseases associated with aging and lifestyle, such as diabetes [70]. This metabolite was also tested in molecular simulations to verify if it was capable of blocking alveolar ACE2 receptors. In the Absorption, Distribution, Metabolism, and Excretion (ADME) assays, the metabolite was found to have an acceptable molecular mass for oral absorption, a good ability to permeate the cell membrane, low aqueous solubility, and a tendency to be absorbed through the gastrointestinal membrane. However, it presented a low capacity to inhibit its receptor, based on the result of its Gibbs free energy (−5.6 Kcal/mol) [71].

### 3.7. Gyrophoric Acid

Gyrophoric acid is a depside commonly found in lichens of the *Umbilicaria* genus, to which different biological activities are conferred [7].

Regarding its anti-proliferative activities, this metabolite is known to exert variable activities on some cancer cell lines. Kosanić et al., 2014, reported that gyrophoric acid was weak against lung and colon cancer cell lines but had moderate cytotoxic activity against melanoma and myelogenous leukemia cell lines [72]. Bačkorová et al., 2012, evaluated the activity of gyrophoric acid against ovarian and colon cancer cell lines. The results obtained were variable: the metabolite was able to cause apoptosis (determined by annexin V staining) in both cancer cell lines; however, these effects were achieved only at its highest concentration (200 µM), and there were differences between the time at which significant results were obtained, with 24 H for ovarian cancer and 72 H for colon cancer. It was also observed that gyrophoric acid decreased MMP only in ovarian cancer cell lines, and it caused an increase in ROS only in colon cancer cells. The metabolite was also able to modulate the expression of the proteins associated with apoptosis (p53, p38, and Bcl-2), also at the highest concentration for most of them. The same authors examined the activity of gyrophoric acid against other cell lines, and it was able to exert cytotoxicity against promyelocytic leukemia, lymphoid leukemia, and ovarian cancer; however, it only acted significantly against three of the nine cell lines tested, and only at high concentrations [54]. Gyrophoric acid also achieved dose- and time-dependent anti-proliferative effects in cervical cancer cell lines, where a decrease in MMP was also observed, as well as the activation of caspase-3, and an increase in ROS after administration of the metabolite, conferring anti-proliferative and proapoptotic activity to gyrophoric acid by induced oxidative stress. Oxidative stress was determined to cause damage at the deoxyribonucleic acid (DNA) level through the 8-oxoguanine biomarker, validated through a separate trial with N-acetylcysteine pretreatment, where it managed to protect cell lines from damage [73].

Based on DPPH assays, gyrophoric acid achieved significant antioxidant activity, with IC_50_ values of 105.75 µg/mL. The metabolite was also shown to have high reducing activity and high superoxide anion inhibitory activity (IC_50_ of 196.62 µg/mL) [72]. Gyrophoric acid also proved to be a good UV filter, with activity that exceeds the reference compound homosalate, based on in vitro determination of sun protection factor (SPF), where a value of 5 was obtained for the metabolite and 4 for homosalate. Furthermore, gyrophoric acid was found to be a good UVB sunscreen [59]. One trial wanted to determine the photoprotective and cytotoxic activity of gyrophoric acid with cell lines derived from HaCaT keratinocytes, and it was found that at concentrations of 400–800 µM, it exerted cytotoxicity, but at sub-toxic doses, it achieved a better photoprotective effect. The metabolite also prevented the harmful effects after UVB irradiation in these cell lines in a dose-dependent manner, considering a concentration of 100 µM as optimal for this activity. Another study revealed that gyrophoric acid also has anti-aging effects on human dermal fibroblasts under treatment with UVA radiation. In the assay, the metabolite was found to be able to increase the expression of *COL1A1* and *COL3A1* transcripts. These are associated with the production of type I collagen, which also increases with gyrophoric acid treatment. In addition, it was found that it also decreased the expression of matrix metalloproteinases, proteins that degrade mature collagen molecules during the aging process [74].

Schinkovitz et al., 2018, conducted a trial using gyrophoric acid and other metabolites to assess their vasodilatory abilities. Using Wistar mice for mesenteric artery testing, capillary wall tension was quantified after adding different concentrations of the metabolite to obtain a mean effective dose (ED_50_) value. In the results, gyrophoric acid was able to exert vasodilation, with an ED_50_ of 1.46 µM. However, the mechanisms involved in this activity were not further investigated [70].

### 3.8. Fumarprotocetraric Acid

Fumarprotocetraric acid is a depsidone found in species from the genus *Lecanora, Cladonia,* and *Usnea*, and it is a metabolite that also has different documented biological activities [3,63].

Regarding the anti-proliferative activities of fumarprotocetraric acid, the extract of the lichen species *Cladonia rangiferina* presented good cytotoxic activity against human melanoma and colon cancer cell lines, with results of IC_50_ of 19.97 and 10.97 µg/mL, respectively. Fumarprotocetraric acid was isolated from these species, but the metabolite itself had lower activity when compared to the extract (IC_50_ of 30–41 µg/mL, approximately). Nevertheless, the molecular mechanism of this metabolite was studied, and it was found that it caused cell cycle arrest in the G0/G1 phase [17]. There are also studies of the extract of *C. islandica* in hepatic and breast cancer cell lines, and the results of IC_50_ were 181.05 and 19.51 µg/mL, respectively [75].

In relation to antioxidant properties, the study of Kosanić et al., 2014, showed that fumarprotocetraric acid has significant antioxidant activity, based on DPPH (IC_50_ of 228.46 µg/mL) and superoxide (IC_50_ of 387.57 µg/mL) radical scavenging assays, as well as reducing power assays, but this activity was not superior to the metabolite atranorin, which was also evaluated in this study [17]. Fernández-Moriano et al., 2015, found that fumarprotocetraric acid is one of the main compounds found in the *C. islandica* extract. After testing the extract, it was found that it could significantly revert cell toxicity induced by hydrogen peroxide in human astrocytoma cell lines, with concentrations that did not affect the cell viability (25 µg/mL). The extract also managed to inhibit the intracellular ROS production induced by hydrogen peroxide, increase the production of the antioxidant enzyme glutathione, and inhibit lipidic peroxidation in these cell lines. The same authors also performed in vitro studies of the isolated metabolite in astrocytoma and neuron-like cell lines to evaluate their neuroprotective properties. Alongside this, antioxidant properties were tested via oxygen radical absorbance capacity (ORAC) assays and DPPH radical scavenging assays, where the results were favorable. Regarding the neuroprotective results in the cell lines treated with hydrogen peroxide, it was found that the pretreatment with 1 µg/mL of fumarprotocetraric acid significantly reduced the damage induced with peroxide and increased the cell viability. Besides this, the metabolite was capable of preventing the peroxide-induced dissipation of the MMP, diminishing the oxidative damage-induced apoptosis, and inducing the expression of various antioxidant enzymes [76].

As for inhibitory properties, the metabolite managed to have inhibitory activity against AGEs, but this activity was not superior to the other metabolites evaluated [70]. Brandão et al., 2017, tested the capacity of fumarprotocetraric acid to inhibit the tyrosinase enzyme, which is important in melanin production. This activity has been considered to be applicated to skin whitening. The metabolite proved to act as a non-competitive inhibitor of tyrosinase, performing its effect in a concentration-dependent way [77]. One study performed in vivo assays with albino Swiss mice to evaluate the expectorant and mucolytic activities of fumarprotocetraric acid. After oral, intraduodenal, and intraperitoneal administration of the metabolite in the animal models, it was found that the compound managed to act in a concentration-dependent way, determined by the increase in the activity of red phenol indicator in the bronchoalveolar lavage. A reduction in lipid peroxidation in the proportion of the tested concentration was also observed, also conferring an antioxidant activity [78]. Microtubule-associated tau proteins are widely involved in Alzheimer’s disease. Under stress factors, this protein detaches from the microtubules and forms aggregates in the soma and the dendrites of the neuron. The group of González et al., 2021, determined that fumarprotocetraric acid can reduce the aggregation of the tau proteins through interaction with the cysteine residue, which is involved in the polymerization [79].

### 3.9. Protolichesterinic Acid

Protolichesterinic acid is an aliphatic acid commonly found in the species *C. islandica* and also in other cetrarioid lichens. Like usnic acid, it has two enantiomers, with (-)-protolichesterinic acid only reported in the species *C. ericetorum* [15].

Brisdelli et al., 2012, examined the cytotoxic properties of protolichesterinic acid obtained from *C. aculeata*, species found in Chile; and this metabolite achieved significant effects on breast, cervical, and colon cancer cell lines at 25 µM of concentration. It was found, testing against HeLa cervical cell line, that protolichesterinic acid increased progressively and significantly the activity of caspase-3, leading to cellular apoptosis [32]. A similar result was observed in the study of Russo et al., 2012, where the compound was isolated from another species present in Chile; *Rhizoplaca melanophthalma*. In this study, it was also observed that the metabolite modulates the expression of the Bax and Bcl-2 proteins, as well as the molecular chaperone HSP70, associated with oncogenesis in cancer cells where its expression is abnormally high [80]. The cytotoxic activity of the crude extract of *Tuckermannopsis ciliaris* in Burkitt lymphoma cell lines was also reported, where protolichesterinic acid was found in the extract by thin layer chromatography [81].

In another study, the metabolite proved to have anti-proliferative and proapoptotic activity against breast cancer cell lines by inducing stress in the endoplasmic reticle, possibly by a disruption of the lipidic metabolism [82]. Years later, the same authors performed more tests in these cell lines, and it was suggested that the metabolite could act by inhibiting the activity of the type-1 fatty acid synthase (FASN) and the expression of the human epidermal growth factor receptor 2 (HER2), involved in the MAPK signaling pathway [83]. The same group performed more studies on anticancer potential, this time using multiple myeloma and pancreatic cancer cell lines, based on reports that protolichesterinic acid inhibited the lipoxygenases, involved in carcinogenesis. However, their effects were not associated with this activity, and they were due of G1 cell cycle arrest and induction of apoptosis (except in pancreatic cancer) [84].

Other studies evaluated the activity of protolichesterinic acid and its synergy with doxorubicin in cervical cancer, neuroblastoma, and myeloid leukemia cell lines [85]. In the results, synergy was only found in cervical cancer cell lines, but in the other two cell lines, only an additive effect was observed. On its own, protolichesterinic acid only caused anti-proliferative effects in concentrations superior to 20 µM. It was determined that the metabolite caused proapoptotic effects by caspase activation. In this study, the metabolite was also isolated from the specie *C. aculeata*.

### 3.10. Physodic Acid

Physodic acid is a depsidone found in the species *Hypogymnia physodes* and *Pseudevernia furfuracea* [63].

Several experimental studies were documented regarding the anti-proliferative properties of physodic acid. Its significant cytotoxic activity has been reported in colon and melanoma cancer cell lines, as well as reports of activity on cervical, hepatic, breast cancer, and glioblastoma cell lines, both after 48 and 72 H of treatment. The results of IC_50_ of these studies are found in Table 2 [86,87,88,89,90].

Regarding the molecular mechanisms of these activities, the reports differ depending on the type of assay and the cancer cell line affected. Researchers found using flow cytometry that in melanoma and colon cancer cell lines there was a large proportion of cells in the sub-G1 apoptotic phase, conferring physodic acid the ability to cause cell cycle arrest in these cancer cell lines [86]. Another study reported that physodic acid was capable of modulating the expression of genes regulated by β-catenin, with *Axin2*, *BIRC5,* and *MMP7* genes as examples [91]. These are associated with cell survival, proliferation, and migration, and are expressed due to the increase in the β-catenin activity caused by the aberration of the Wnt signaling pathway in the tested colorectal cancer cell lines. Emsen et al., 2020, observed in their study with hepatic cancer cell lines that physodic acid caused an increase in the total oxidative status, leading to oxidative stress in these cells [89]. Alongside this, it was found that the metabolite also modulated the expression of proteins associated with apoptosis (caspase-3, Bcl-2, Bax, among others). This protein modulation ability was also observed in a study with a melanoma cell line [33].

The metabolite showed significant antioxidant activity in a DPPH radical scavenging assay, obtaining a value of IC_50_ of 69.11 µg/mL. Alongside that, in a superoxide anion assay, it managed to significantly scavenge this radical, and also proved to have a high reducing power [86]. It was also found that physodic acid in low concentrations (6.25–25 mg/L) granted an antioxidant capacity in human fibroblast cultures [92]. A similar effect was observed in hepatic cell lines, with a concentration-dependent increase in the total antioxidant capacity [89].

Different depsides and depsidones were tested in their potential to inhibit the microsomal prostaglandin E2 synthase-1 (mPGES-1), which is the target of a novel mechanism of action that sought to achieve an anti-inflammatory effect that does not cause adverse effects associated with the inhibition of the COXs [93]. Physodic acid was one of the metabolites that were similar to one of the two pharmacophore models that had inhibitory potential against mPGES-1. In order to corroborate this finding, an assay was conducted using the microsomal fractions of A459 cell lines stimulated with IL-1β interleukins, using prostaglandin H2 (PGH2) as substrate. The activity of physodic acid was confirmed since it managed to inhibit the conversion of PGH2 to prostaglandin E2 (PGE2).

This metabolite has proved to be capable of inhibiting some medically relevant enzymes. One of these is hyaluronidase, an enzyme that degrades hyaluronic acid in the brain extracellular matrix, a process that is associated with increased cancer cell proliferation. This compound can also inhibit the butyrylcholinesterase enzyme associated with neurodegeneration. The study that determined this inhibitory activity with both previous enzymes also found via an artificial membrane permeability assay for the blood-brain barrier (BBB) that physodic acid has an acceptable permeability coefficient and can easily cross the BBB [90].

### 3.11. Diffractaic Acid

Diffractaic acid is a compound part of the depside group that has been isolated from species from the genus *Usnea* and the native species *Protousnea magellanica* [63]. Several of the studies to be mentioned used diffractaic acid isolated from this species.

There are many reports regarding the anticancer potential of diffractaic acid. Brisdelli et al., 2012, tested its effects in breast, cervical, and colon cancer cell lines. In the results, the metabolite showed remarkable activity in the colon cancer cell lines from 25 µM, but only at concentrations higher than 50 µM did it cause effects on the other two cancer cell lines. The results of the IC_50_ were between 42.2 and 93.4 µM, which is considered moderate in comparison to other tested metabolites like usnic acid [32]. An intermediate activity was also reported in melanoma cell lines [94]. The group of Russo et al., 2012, performed a study of its activity against prostate cancer cell lines. In this case, the metabolite only caused significant effects in concentrations between 25 and 50 µM, considering it a low cytotoxic activity. It is because of this low activity that in this work that no further tests were carried out to determine its molecular mechanism [80]. Researchers tested the anticancer capacity of diffractaic acid on in vivo assays [95]. In Swiss mice, Ehrlich ascites carcinoma (EAC) cell lines were inoculated via intraperitoneal. The results show that the metabolite caused a growth reduction in the EAC cells in a dose-dependent manner. The metabolite also exerted protective effects on the tissues of the liver, kidneys, and small intestines in doses of 50 mg/Kg, reducing the inflammation and the necrotic area caused by EAC. However, it was also observed that diffractaic acid reduced the platelets count and hemoglobin in the blood, especially on higher doses. Another group tested the activity of diffractaic acid in glioblastoma multiforme. The results showed that at high concentrations (from 40 mg/L), the metabolite managed to inhibit cell proliferation. These same concentrations caused a high total oxidative status in these cells. The value obtained of IC_50_ for diffractaic acid in the glioblastoma cell lines was 35.67 mg/L [65].

Diffractaic acid proved to have certain antioxidant activity in the study of Maulidiyah et al., 2020, wherein the DPPH assay displayed an IC_50_ value of 18.51 µg/mL. Nevertheless, this value was not superior to the value of the positive control quercetin. This metabolite also showed a remarkable total antioxidant capacity in concentrations of 10 mg/L in primary rat cerebral cortex cells [96].

In mention of anti-inflammatory effects, diffractaic acid was capable of inhibiting the development of indomethacin-induced gastric ulcers in in vivo studies with mice. Concentration-dependent activity was observed regarding a reduction in gastric ulcers, and it was found that the metabolite increased the expression of antioxidant enzymes and decreased the lipid peroxidation and also neutrophile infiltration [97]. However, no recent studies were found regarding this activity.

The hepatoprotective capacity of diffractaic acid was evaluated in in vivo studies. Wistar albino mice were used, to which hepatic damage was induced via carbon tetrachloride intraperitoneally administered daily for 6 weeks. In the biochemical results, it was found that diffractaic acid mitigated the increase in hepatic enzymes aspartate aminotransferase (AST), alanine aminotransferase (ALT), and gamma-glutamyl transferase (GGT) at doses of 50 mg/Kg, while higher doses increased these hepatic enzymes. In the histological analysis, this same dose of diffractaic acid reduced the tissular damage caused by carbon tetrachloride, as well as the degree of inflammation [98].

### 3.12. Salazinic Acid

Salazinic acid is a compound that belongs to the depsidones group, present in lichens of the genus *Xanthoparmelia*, *Parmelia*, among others [63].

Some anti-proliferative activities against cervical, lung, colon, melanoma, and Burkitt lymphoma cancer cell lines were documented, but these results came from studies with the crude extracts of lichens that contained salazinic acid, so their activity could be associated with other metabolites apart from the aforementioned compound [81,99,100,101]. Manojlović et al., 2012, performed cytotoxic assays against melanoma and colon cancer cell lines, and the results of the IC_50_ for the isolated metabolite were 39.02 and 25.67 µg/mL, respectively. It was found that salazinic acid caused a cell cycle arrest in the sub-G1 apoptotic phase [102]. The group of Alexandrino et al., 2019, also made cytotoxicity tests with salazinic acid, and in their in vitro assays, it was found that the metabolite had a significant anti-proliferative activity on human colon, prostate, and myelogenous leukemia cell lines, as well as murine melanoma cell lines. Although a significant increase in the number of apoptotic cells was observed, this action could not be associated with caspase-3 activation. The metabolite also inhibited the tumoral growth of murine melanoma cell lines on in vivo assays with BALB/c mice [103].

Various studies regarding the antioxidant activity of salazinic acid have been documented. Some of them were made using crude extracts of lichen where the metabolite is present, such as the work of Amo de Paz et al., 2010, where the extract of *Xanthoparmelia camtschadalis* exerted an antioxidant capacity superior to the isolated metabolite, based on the ORAC assay and cell viability assays using astrocytoma cell lines. In this work, both the lichen extract and salazinic acid was found to reduce cell viability in these cell lines at 250 and 50 µg/mL of concentration, respectively, but the pretreatment of 5 and 10 µg/mL of the isolated metabolite protected this cell line from the hydrogen peroxide treatment, significantly increasing the percentage of cell viability in comparison to the control group [104]. Another study tested the extracts of *Parmelia sulcata* and *P. saxatilis*, which also contained salazinic acid, and between these species, there were favorable but differential results regarding their DPPH radical scavenging capacity and total flavonoid content [99]. This difference in activity between extract and isolated metabolite can be attributed due the presence of other antioxidant compounds in the crude extract, a detail that has been addressed in other studies where metabolites such as usnic acid, atranorin, norstictic acid, and others were found [101]. Manojlović et al., 2012, made tests with the isolated metabolite and found that it exerted a strong DPPH radical (IC_50_ of 91.52 µg/mL) and superoxide anion (IC_50_ of 138.23 µg/mL) scavenging activity [102]. Similar results were observed in the study of Selvaraj et al., 2015, with a concentration-dependent activity towards the hydroxyl and superoxide radicals, as well as the inhibition of lipid peroxidation [105]. Salazinic acid also proved to be a good candidate as a UVA protection factor booster [59].

### 3.13. Other Secondary Metabolites

There are several other lichen secondary metabolites with documented biological activities. However, the studies associated with these activities are not relatively recent, so there is little up-to-date information in this regard [1]. Furthermore, these less investigated metabolites have been analyzed in the majority of studies on lichen extracts and not in their isolated form; therefore, said activity can be caused by a joint effect of several compounds [100,101]. Thus, the most relevant discoveries regarding the biological activities of other lichen metabolites are addressed.

Ebrahim et al., 2016, tested the anti-proliferative properties of norstictic acid, isolated from the species *Usnea strigosa*, in breast cancer cell lines. In the results, the metabolite managed to significantly inhibit cell proliferation in a concentration-dependent manner, with a higher effect in two cell lines known for overexpressing the receptor tyrosine kinase c-Met, which its affinity with the hepatocyte growth factor (HGF) promotes its aggressive growth and metastasis induction. The study also wanted to determine if the metabolite caused cell death or cell cycle arrest, and through the LDH assay, it was observed that its effects consisted of the arrest of the cell cycle instead of the induction of cell death, proved by the lack of LDH increase. It was also observed that norstictic acid reduced the cell migration and invasion on in vitro assays and also that the metabolite acted selectively with cancer cells after tests with normal mammary epithelial cell lines with no significant results. Through molecular docking studies, it was found that c-Met could be a potential target for the activity of norstictic acid. Finally, the metabolite effects were corroborated on in vivo assays with nude mice, where significant tumor suppression was observed [106].

One study evaluated various lichen secondary metabolites regarding their neurotrophic activities on Neuro2A murine neuroblastoma cell lines in order to explore their potential as central nervous system therapeutic agents. Although metabolites like atranorin, physodic acid, and usnic acid managed to significantly increase the neurite growth at concentrations of 5 µM, the metabolite perlatolic acid exerted the same activity but with 0.5 µM of concentration; in addition to not causing cytotoxic effects, together with atranorin and physodic acid. It was also observed that the metabolites atranorin, physodic acid, and perlatolic acid induced the increase in the neurotrophic genes of nervous growth factor (NGF) and brain-derived neurotrophic factor (BDNF). Additionally, perlatolic acid showed higher neurotrophic activity than the standard neuroactive compound resveratrol. After seeing that perlatolic acid exerted a neurotrophic activity with a lower concentration, neurosphere assays were performed from a mice hippocampi culture, where the metabolite managed to significantly increase the proliferation of neural stem/progenitors cells at the same concentration that exerted neurotrophic effects. The group wanted to determine if perlatolic acid was capable of inhibiting the enzyme AChE, and the metabolite achieved this activity with a result of IC_50_ of 6.8 µM, compared to the standard galantamine (IC_50_ of 2.5 µM). Finally, the molecular mechanism of the neurotrophic activity of perlatolic acid was evaluated, and it was found that the metabolite increases the acetylation of the H3 and H4 histones, a modification involved in the neural stem/progenitors cells differentiation [107].

Secondary metabolites isolated from lichen species found in Chile, such as physciosporin and tumidulin, were mentioned above. In a study with lung cancer cell lines, the metabolite physciosporin, present in the extract *Pseudocyphellaria coriacea*, managed to inhibit the cell migration at a concentration of 5 µg/mL, along with inhibiting its invasive capacity in Boyden chamber assays. It was found later on that the metabolite was capable of modulating the expression levels of epithelial-mesenchymal transition (EMT) markers at the messenger ribonucleic acid (mRNA) level; which facilitates cell motility; alongside the suppression of the KITENIN-mediated AP-1 activity and the reduction in the Rho-GTPase activity; thus, supporting the antimetastatic activity of the metabolite [34]. A similar activity was observed with colon cancer cell lines, where the IC_50_ values obtained were between 11.5–29.7 µg/mL in the cell viability assays. It was also found that physciosporin increased the levels of caspase-3 and the cleaved PARP, associating its activity with apoptosis induction. In non-toxic doses, the metabolite suppressed the cell motility, as well as its tumorigenic potential. Alongside the aforementioned molecular activities, physciosporin also reduced the cell motility associated with actin and mediated by the vascular endothelial growth factor A (VEGFA), whose expression was reduced along with other associated genes, conferring its metastatic potential as well [108]. In a study with breast cancer cell lines, it was proved that besides exerting a concentration-dependent cytotoxicity, cell cycle arrest in the sub-G1 phase, and the inhibition of the tumorigenic potential, physciosporin suppressed the level of the transcriptional factors β-catenin, cyclin D1 and c-Myc, as well as HIF-1α and NF-kB; which play a role in carcinogenesis, mitochondrial respiration and the mechanism of aerobic glycolysis, which is used to give energy to cancerous cells [109]. It is also worth mentioning the metabolite epanorin, isolated from the species *Acarospora schleicheri* recollected from the alpine zone of the north of Chile. With this metabolite an in vitro study was performed with breast cancer cell lines, which managed to inhibit cell proliferation, achieving the maximum inhibition percentage (~80%) with a concentration of 28 µM, based on sulforhodamine-B cell proliferation assays. It was found that epanorin caused a significant cell cycle arrest in the G0/G1 phase [110].

The metabolite physciosporin was also tested against cancer stem cells, theorized to be responsible for tumoral persistence and failure of anticancer therapies. According to this theory, they possess a self-renewal capacity similar to that of common stem cells; they are also resistant to apoptosis and can give rise to a new progeny with a variable proliferative potential. Therapeutic advances seek to block signaling pathways such as Notch and Hedgehog, which play a role in the development of these cancer stem cells. The metabolite physciosporin, isolated from *Pseudocyphellaria granulata*, exerted a significant cytotoxic activity at a concentration of 50 µg/mL against colorectal cancer stem cell line CSC221; however, the crude extract achieved a higher activity at the same concentration. In addition, both extract and metabolite managed to significantly decrease the colony and spheroid formation in a dose-dependent manner. It was also observed that both were able to inhibit the stemness potential of colorectal cancer stem cell lines by reducing the formation of spheroids of these cell lines from a concentration of 5 µg/mL. This was validated by observing that in the CSC221 cell lines, the expression of proteins and genes associated with this activity was suppressed, such as aldehyde dehydrogenase-1 (ALDH1), Musashi-1, Hes1, and Gli2, among others. With the purpose of corroborating and investigating the role of physciosporin in the signaling pathways mentioned before, assays with embryonic kidney cells were performed. The activity of Hes1, a gen directed to the ligands of the Notch pathway, was reduced with the action of the extract and metabolite at concentrations of 5–10 µg/mL, as well as the activity of the genes *Gli* and *CSL*, main transcriptional factors of the Sonic hedgehog pathway (SHH) and Notch signaling pathways, respectively. All of this suggests that the Notch and SHH signaling pathways are involved in the anticancer activity of physciosporin. This was confirmed in a last spheroid assay with the CSC221 cell lines, which overexpresses these genes, where the metabolite managed to suppress the spheroid formation in a dose-dependent manner [35]. A similar activity was observed with the metabolite tumidulin, isolated from the species *Niebla* sp., where, in the study, it was reported that both metabolite and extract reduced the spheroid formation of CSC221 and colorectal cancer cell lines, in addition to modulating the expression of genes associated with Hedgehog signaling pathways, such as the smoothened proteins (SMO) and the ones from the Gli family; thus, reducing the stemness potential of the cancer cell lines [36].

There are numerous reports of biological activities of other, lesser-known secondary metabolites of lichens, among which are some of the compounds mentioned before. In summary, the remaining secondary metabolites, their documented biological activities, as well as the species where these compounds were isolated are presented in Table 3.

## 4. Pharmacological and Toxicological Considerations

If the possible effects in the body of these lichen secondary metabolites were to be evaluated, we must bear in mind different pharmacological considerations, mainly the toxicity that the compounds could exert in the body, as well as pharmacokinetic and pharmacodynamic parameters. Although it was noted that there are no clinical trials regarding lichen secondary metabolites, there are other studies, mainly in vivo trials, which explored these previous areas, especially the toxic effects of these metabolites. It is known that lichens and their substances can be allergenic and cause hypersensitivity reactions in people that are susceptible. According to the information compiled by Molnár and Farkas, the metabolite atranorin, diffractaic acid, evernic acid, fumarprotocetraric acid, lobaric acid, perlatolic acid, physodic acid, salazinic acid, and usnic acid possess reports of allergic reactions, including contact dermatitis, scaling, rhinitis, and asthma [3]. Despite this, allergy to lichens in people is considered uncommon, and the potency of its hypersensitivity effects ranges from weak to moderate [37].

Concerning usnic acid toxicological effects, it has been shown that toxicity reports in wild animals, such as the case of approximately 500 elk that died in a 7-week episode in early 2004, in which symptoms of paralysis, muscle weakness, and ataxia were observed. The cause of death was identified as due to the ingestion of the lichen *Xanthoparmelia chlorochroa*, which contains 1–2% of usnic acid. A group of researchers carried out an in vivo study based on this case, using domestic sheep as test models, which were administered 102 mg/Kg/day of usnic acid with the food, and it was found that the metabolite caused histological alterations to the skeletal muscle, concluding that the metabolite caused myotoxicity in ruminant animals. However, this same study also mentions that usnic acid cause hepatotoxicity in monogastric animals [119]. There are in vivo studies with Swiss mice that, when treated with 15 mg/Kg of usnic acid intraperitoneally for 15 days, caused an increase in transaminases and extensive hepatic necrosis, although no apparent toxicity was observed. Another study with Wistar mice using doses of 50–200 mg/Kg of usnic acid intraperitoneally for 5 days showed that it caused swelling in the hepatic mitochondria and endoplasmic reticulum, associated with mild hepatic damage [120]. An in vitro study with murine hepatocytes revealed that in concentrations of 5 and 10 µM, the cell viability of these cells was significantly reduced as the exposure time increased, and that the adenosine triphosphate (ATP) content was reduced in a similar manner [121]. The median lethal dose (LD_50_) values for intravenous administration in animals was 25 mg/Kg in mice and 30 mg/Kg in rabbits [38], while the oral LD_50_ value was 838 mg/Kg in mice and >500 mg/Kg in rabbits [122].

There are also cases of usnic acid toxicity in humans, mainly associated with the dietary supplement LipoKinetix, manufactured by Syntrax, which was commercialized as a weight loss product. A total of 100 mg of sodium usnate is included in its composition, the salt form of usnic acid, and according to the manufacturer, the supplement possesses a supposed mechanism of action that claims to “imitate exercise” by “modifying the process in the body called oxidative phosphorylation”. The recommended dosage of the product was 1–2 capsules three times a day, which gives results to a dose higher than that established in the information found in Traditional Chinese Medicine for usnic acid, which is approximately 60–120 mg/day [120,123]. The work of Favreau et al., 2002, presented the case of 7 previously healthy patients that developed severe hepatotoxicity while consuming LipoKinetix during the year 2000. Five of the patients were taking the product for 1 month or less, while the other two were for 2 to 3 months. Signs and symptoms that the patients developed included nausea, vomiting, fatigue, abdominal pain, an increase in transaminases, jaundice, and a case of fulminant hepatic failure and hepatic encephalopathy that required intubation and treatment with mannitol. All the patients recovered spontaneously after discontinuation of the supplement, with hepatic tests normalized in five patients under observation after 4 months [123]. According to a document presented in the year 2005 by the National Toxicology Program of the United States, in collaboration with the Food and Drug Administration (FDA), at least 21 reports of adverse effects associated with LipoKinetix have been received, including one case of death, one case of a liver transplant, seven cases of hepatic failure, ten cases of chemical hepatitis, and four cases of acute hepatotoxicity [122]. After the FDA issued a public warning regarding this product, LipoKinetix was withdrawn from the market in November 2001 [124].

Many researchers suggest that the hepatotoxic mechanism of usnic acid is due to its potential to uncouple oxidative phosphorylation in the mitochondria. This activity causes the inhibition of the electron transport chain, which could lead to a decrease in ATP synthesis [122,123]. One theory proposes that, due to its fat-soluble nature, usnic acid and its salt form can pass through the inner mitochondrial membrane by passive diffusion into the mitochondrial matrix, where the compound is ionized and releases a proton in the matrix. The resulting usnate anion is capable of diffusing back to the intermembrane space, to re-form usnic acid after binding to a proton present in the acidic side of the inner-membrane proton gradient, thus allowing it to diffuse to the matrix. Due to this cycle, a proton leakage occurs that will dissipate the proton gradient across the inner membrane, disrupting the coupling between electron transport and ATP synthesis. It is also believed that the antimicrobial activity of usnic acid is due to this mitochondrial mechanism [120]. This activity has been demonstrated in several studies, where concentrations greater than 6 µM of usnic acid managed to exert the uncoupling effect of oxidative phosphorylation with greater potency in murine hepatocytes [121,125]. Moreira et al., 2013, conducted a study in a murine liver perfusion system with the goal of investigating the metabolic effects of usnic acid in said organ. Under concentrations between 1 and 10 µM, it was observed that usnic acid stimulated oxygen consumption in low concentrations, reduced the ATP content, inhibited gluconeogenesis, stimulated glycolysis and fructolysis as a compensatory mechanism for the low mitochondrial ATP production, and also stimulated glycogenolysis in order to increase the glycolytic flow, among other harmful metabolic phenomena [126]. All the aforementioned effects are seen in compounds that exert oxidative phosphorylation uncoupling, noticing that usnic acid exerts these effects over short ranges of concentration, such as those mentioned above. Mechanisms other than uncoupling capacity are also associated, such as the induction of oxidative stress in murine hepatocytes [127].

Regarding the pharmacokinetic and pharmacodynamic properties of lichen secondary metabolites, the situation is similar to the toxicologic information available to date, where usnic acid is the only compound that has studies related to these characteristics. There are in vivo studies with rabbits where 5 and 20 mg/Kg of usnic acid were administered intravenously and orally, respectively. Considering the period of time of these works (1993–1995), we present the most relevant results mentioned in more recent reviews. After intravenous treatment, the value of terminal half-life was approximately 10.7 H, and the value of total clearance was 12 mL/H/Kg and also had plasma concentrations levels superior to 8 µg/mL for more than 12 H after administration [128]. Regarding the oral treatment, the value of the half-life was approximately 18.9 H, with a maximum plasma concentration of 32.5 µg/mL after 12 H. The oral bioavailability obtained was 78% [120,129]. Another first-time study wanted to determine the protein binding capacity of usnic acid in rabbit plasma and bovine serum albumin (BSA) alongside tissue distribution studies via in vivo assays with Wistar mice, which were administered 20 mg/Kg of the metabolite intraperitoneally. In the results, usnic acid showed a high protein binding capacity of 99.2%, which was dependent on the albumin concentration, proved after a reduction in the protein binding capacity in lower concentrations, although the binding capacity was constant with 6.5 g/L of albumin. Regarding its tissue distribution, it was observed that there were higher concentrations of usnic acid in the lung, liver, and blood, remaining constant after 6 H, while fat tissue and brain showed lower concentrations of the metabolite [130].

Finally, usnic acid was evaluated in a metabolism study on plasma, hepatocytes, and human liver subcellular fractions. The result obtained for protein binding in human plasma was 99.76%, with a concentration of 1 µM of usnic acid. Alongside this, 30 µM of the metabolite was incubated in S9 human liver subcellular fractions, together with the reduced form of nicotinamide adenine dinucleotide phosphate (NADPH) and uridine 5′-diphosphoglucuronic acid (UDPGA) for 45 min. By using liquid chromatography-mass spectrometry, three oxidized metabolites and two glucuronide conjugates were identified. In human liver microsomes, the half-life of the metabolite was 19.3 min, and the intrinsic clearance was approximately 45 mL/min/Kg, mediated by phase I metabolism after seeing that the clearance value was similar to the one obtained in the microsomes fortified only with NADPH. It was also determined that the cytochrome P450 CYP1A2 was involved in the phase I metabolism of usnic acid and that the UGT1A1 and UGT1A3 catalyzed the formation of both glucuronide conjugates, with a minor contribution of UGT1A8 in higher concentrations of the lichen metabolite. It was also determined that usnic acid was an inhibitor of CYP2C19 and CYP2C9, with IC_50_ values of 1.9 and 6.3 µM, respectively [131].

## 5. Discussion

The biological activities of a total of 26 lichen secondary metabolites were found and described using our search parameters, mainly from compounds found in species of the genus *Cetraria, Cladonia, Cornicularia, Hypogymnia, Lecanora, Ochrolechia, Parmelia, Parmotrema, Protousnea, Pseudevernia, Psoroma, Ramalina, Rhizocarpon, Sphaerophorus, Stereocaulon, Umbilicaria,* and *Usnea*. The most exceptional secondary metabolites belong to the groups of dibenzofurans, depsides, depsidones, and aliphatic acids, and the most frequently described biological activities were the anticancer, antioxidant, and anti-inflammatory activities. Many of the characteristics presented in each secondary metabolite provide promising results in terms of their biological activity, and which of these metabolites is capable of exerting said activities with a better potency should be evaluated next; that is, at which concentration they exert their optimal effects, ideally if this concentration is lower [132] to avoid any unwanted side effects or negative responses in a future pharmaceutical use.

It is evident that the dibenzofuran usnic acid is the metabolite that has better support in both its biological activities and its effects on the body with a pharmaceutical purpose. This is because it is one of the most studied secondary metabolites, mainly due to the high content in some lichens and its wide distribution between species [37]. However, this metabolite has a varied number of documented biological activities, such as anticancer, antimicrobial, antioxidant, anti-inflammatory, wound-healing, antiviral, and photoprotective, among others. After usnic acid, atranorin would be the second most studied lichen metabolite [54]; however, it lacks toxicological studies and has much fewer clinical trials. As for the rest of the metabolites mentioned in this work, all of them possess varied biological activity studies, which decrease in number in correlation with the little available biological information that is known about these species. It is also a factor that there are few recent studies on these lesser-known metabolites, to the point that it was necessary to resort to older information in order to know their biological activities [97,113]. However, this could be due to the fact that the activities described at the time of certain compounds did not deliver promising results, limiting the possibility of further studies regarding their biological activities or interest in future studies with these metabolites. Despite this, it is worth highlighting some relevant discoveries on secondary metabolites that exert biological activities of current importance.

Within the anticancer potential of the secondary metabolites of lichens, several molecular mechanisms were found that cause cytotoxic effects in cancer cells; many of these with the purpose of triggering cell apoptosis, such as the depolarization of the mitochondrial membrane potential, the modulation of genes and proteins that regulate apoptosis (such as p53, Bcl-2, Bax, among others), the activation of caspase-3 and the intracellular increase in ROS. Several metabolites were also capable of causing cell cycle arrest in cancer cell lines, mainly in the G0/G1 phase, as well as mechanisms that mitigate the metastatic and tumorigenic potential of some cancer cell lines. The metabolites that have been studied the most in different cancer cell lines are usnic acid, atranorin, gyrophoric acid, protolichesterinic acid, and physodic acid. However, if we see it from the point of view of the potency of the metabolites in exerting their effects in a lower concentration (considering the results of IC_50_ and EC_50_), the most efficient ones were usnic acid [38,42,111], protolichesterinic acid [83,84], and physodic acid [86]. Other metabolites also proved to be as efficient in their anti-proliferative activity, such as norstictic acid [106], and physciosporin [108,109], but they do not have more studies in other different cancer cell lines. If there is something that must be considered to assess the anticancer potential, it is the type of cell that is being attacked, some being more resistant than others, as in the case of gastric cancer [40], lung cancer, and colon cancer [41,54,71], which required higher concentrations of a secondary metabolite to achieve significative effects. Another characteristic to consider is cytotoxic selectivity, that is, the activity only affects cancer cells, and this has been demonstrated in several studies with non-cancerous cell lines, where the metabolites usnic acid, atranorin, lobaric acid, protolichesterinic acid, diffractaic acid, vicanicin [32,38,56,80], gyrophoric acid [72], physodic acid [33], and physciosporin [108] did not exert significant cytotoxicity in said cell lines.

As for antioxidant activity, it is clear that a large part of lichen secondary metabolites has some degree of activity due to their phenolic structure, which is capable of donating a hydrogen atom to a free radical, thanks to its reductive potential, in order to stabilize the radical and make it a neutral molecule [133]. With the exception of usnic acid and diffractaic acid [44,134], metabolites such as atranorin [58], gyrophoric acid [72], fumarprotocetraric acid [17], physodic acid [86], and salazinic acid [105] managed to scavenge different free radicals in relatively high concentrations. However, it was observed that some metabolites at low concentrations achieved protective effects against free radicals in in vitro studies [45,76,92]. Although it was noted that the redox nature of these secondary metabolites is dependent on the system in which it is found, it can be inferred that it could depend on the concentration of the metabolite. In an in vitro study, usnic acid at concentrations of 1–2.5 µg/mL managed to exert antioxidant activity and protect neuron-like cell lines against hydrogen peroxide [45]. However, another study with usnic acid in concentrations of 20 µg/mL caused prooxidative effects and reduced the cell viability of the same neuron-like cell lines [46]. It could be theorized that concentration is a determining factor in antioxidant activity, but there are no other studies with other secondary metabolites that demonstrated this proposal. Although antioxidant activity is associated with the action of phenolic compounds, the induction of the expression of antioxidant enzymes was also observed [45,75,76], which are crucial in maintaining the equilibrium of reactive oxygen species in the body, whose uncontrolled increase leads to the development of several diseases [134].

Another biological activity that was found frequently in lichen secondary metabolites was the anti-inflammatory effect. It was observed in various studies that the compounds that exert this activity were capable of inhibiting the cyclooxygenase enzymes and suppressing pro-inflammatory molecules like cytokines and the TNF-α [49,60,68]. Usnic and lobaric acid showed to be capable of acting against the NF-kB activity [49,69], which is important since it plays a role in regulating the inflammatory response, increasing the expression of cytokines and pro-inflammatory enzymes. Some of these cytokines can directly activate the NF-kB signaling pathway, generating a positive auto-regulatory loop that increases the inflammatory response, leading to the onset of inflammatory pathogenesis and the development of chronic inflammatory diseases [135]. The in vivo studies showed how some metabolites reduced the infiltration of macrophages and neutrophiles, alongside a wound-healing activity, mainly with metabolites like usnic acid and atranorin [51,60]. In addition to their anti-inflammatory effects, various metabolites demonstrated a certain potential to have activity on the central nervous system. Beyond the antioxidant action on cell lines similar to those present in the central nervous system [45,58,76], metabolites like usnic acid [44], lobaric acid [67], and perlatolic acid [107] managed to inhibit the AChE enzyme, which is present mainly in neuromuscular union and the cerebral synapse. In Alzheimer’s disease, there is a loss of cholinergic neurons and the neurotransmitter itself; therefore, the inhibition of the AChE enzyme could help treat symptoms associated with memory and cognitive function [136]. Other remarkable neuroprotective activities also include the action of fumarprotocetraric acid against the tau microtubules [79] and the neurite growth induction activity of some metabolites, but mainly from perlatolic acid [107].

Several lichen secondary metabolites proved to have promising biological effects, and many of these compounds are found in species present in the Chilean territory, both at the Antarctic and at the continental level. Compounds like usnic acid, atranorin, and gyrophoric acid have been identified frequently in various species present in the South Shetland Islands. Other remarkable metabolites, like protolichesterinic acid, lobaric acid, sphaerophorin, rhizocarpic acid, and physodic acid, were found in the lichens *C. aculeata*, *S. alpinum*, *S. globosus*, *R. geographicum*, and *H. lugubris,* respectively [25,27]. These lichens present in these polar territories are part of a wide group of organisms called extremophiles, which are capable of living in adverse environmental conditions that other organisms would not be able to survive. Low temperatures, strong winds, and environments low in nutrients and with high levels of ultraviolet radiation are common in the Antarctic regions, and the organisms present in said regions require many physical and biochemical adaptations in order to thrive in these conditions. Many natural compounds with biological activities have been isolated from different organisms that inhabit these polar regions, where lichen is also included [137]. In one study that carried out a molecular analysis on numerous species of lichens from different environments, using Raman spectroscopy (a non-destructive technique based on laser radiation that analyzes the vibrational spectrum of molecules, resulting in a signal based on the characteristics of a chemical compound), it was found that metabolites such as rhizocarpic acid are found frequently in lichen species present in polar environments, mostly Antarctica. However, there is still doubt if the production of more complex chemical compounds in lichens is associated with these extreme environmental conditions since, although they were found in a greater number of polar species, these compounds were not exclusive to them and have been found in species from other climates [138]. In relation to the above, it is worth mentioning other metabolites of interest that were isolated from lichens present in continental Chile, like vicanicin isolated from *E. chilense* and lichens from the genus *Psoroma*, species found in the southern zone of Chile [26,28], as well as physciosporin and tumidulin, compounds isolated from lichens of the genus *Pseudocyphellaria* and *Niebla*, respectively [34,36]. And one particular case is that of the metabolite epanorin, isolated from the lichen *Acarospora schleicheri* recollected near Enquelga and Isluga in the north of Chile [110]. With these last three metabolites, there are few experimental studies, but they show promising anti-proliferative activities, so they present an opportunity for both national and international research.

With all the biological activities recompiled in this work, various lichen secondary metabolites seem to be good candidates to become new pharmaceutical molecules. Many metabolites exert different molecular mechanisms to carry out their biological effects, mainly in their anti-proliferative activities and the inhibition of certain medically relevant enzymes. Despite this, the exact mechanism of action of some metabolites and their biological activities are still unclear. The mechanism of action is understood as the interaction between a molecule with a specific target, being an enzyme, protein receptor or other biological molecules, with the purpose of starting a biological response, and its identification is considered important during the process of drug discovery. Although a debate exists regarding the true importance of determining the mechanism of action of a new molecule, considering that a fifth part of the drug approved by the FDA does not have a known mechanism of action or target and that other regulatory agencies approve new drugs as long as they are safe and effective in their pharmacologic activity [139]. Still, the identification of the mechanism of action is a relevant point to consider when investigating the biological activities of these secondary metabolites.

If there is something that stops lichen secondary metabolites from being candidates for pharmaceutical molecules is the lack of clinical trials and the scarce toxicological information available for most of these compounds. Despite the fact that usnic acid has in vivo studies that reveal pharmacokinetic and pharmacodynamic characteristics, these works are not entirely recent, although they could be referential for new studies with these same purposes, at least for this metabolite [128,130]. Regarding secondary metabolite toxicity, aside from usnic acid, it is unknown what toxic effects could cause other lichen compounds in the body. The case of LipoKinetix is a perfect example of trying to recklessly market these secondary metabolites as pharmaceutical products without testing the risks of their use before. Some authors propose that the adverse reaction of this product or usnic acid itself is idiosyncratic in nature, associating its harmful effects with one or more risk factors [123,131]. The term idiosyncratic adverse reaction is commonly associated with an adverse reaction that does not occur in all the patients receiving drug treatment, and the susceptibility to these reactions is determined by patient-specific factors, whether they are immunological predispositions, or genetics, among others [140]. Although usnic acid and some lichen extracts have been used in cosmetics, personal hygiene products, sunscreens, and perfumes, in addition to their use in traditional medicine [120], the bases for these compounds to be formulated into pharmaceuticals remain ambiguous so far.

Nevertheless, many efforts have been made to reduce the toxicity of usnic acid, mainly through derivate compounds. In the study of Schinkovitz et al., 2018, where the in vitro capacity of several lichen secondary metabolites and semi-synthetic compounds for the inhibition of AGEs were tested, a synthetically modified molecule of usnic acid significantly inhibited the AGEs formation [70]. In relation to usnic acid, the triketone moiety of the molecule is attributed to the uncoupling activity of the oxidative phosphorylation; and the reaction of its carbonyl group with primary amines produces the enamine-type derivates. In vitro studies showed that these groups of derivates exert lower toxicity in comparison to usnic acid. There are many usnic acid derivates, which are all developed not only to reduce the toxicity of the base compound, but also to improve the physicochemical characteristics of the molecule, to increase the polarity or solubility, and to discover new and interesting biological activities [129,141]. Another technique that has been used to reduce the toxicity of usnic acid is nanoencapsulation. This technology is applied to molecules like peptides, proteins, and anticancer drugs to facilitate their penetration into cells to improve the dissolution of hydrophobic compounds and to protect these molecules from biological fluids. An in vivo study evaluated the anti-proliferative properties of poly lactic-co-glycolic acid polymer (PLGA) nanocapsules that contained usnic acid on mice, and in the results, a tumoral inhibition of approximately 70% for nano-encapsulated usnic acid was observed in comparison to a suspension of the metabolite itself (43% approximately). The histopathological analysis showed that, although morphological alterations were observed in both forms of usnic acid, the nanoencapsulation caused hepatotoxicity to a lesser extent [142]. The application of these nanoencapsulation systems makes it possible to modulate the release of therapeutic doses of an active compound, increasing the efficacy of the treatment and minimizing the adverse effects, in addition to overcoming the physicochemical and toxicological obstacles of said compounds. Usnic acid has already been evaluated in other delivery systems, such as liposomes, cyclodextrins, and magnetic or metallic nanoparticles (for example, silver, copper, and zinc), with favorable results over the free form of usnic acid in antimicrobial, anticancer, and antioxidant studies [143]. Therefore, these nanosystems would provide advantageous alternatives to investigate the biological activities of lichens efficiently and safely.

## 6. Conclusions

In conclusion, lichens produce secondary metabolites with remarkable biological activities that, if further studied, may have the potential to be pharmaceutical molecules. Many of the biological effects described involve varied molecular mechanisms, especially in terms of anticancer activities and inhibitory action on medically relevant enzymes; however, future experimental studies on these metabolites should focus on identifying the possible specific mechanisms of action, especially in terms of their antioxidant and anti-inflammatory activity, in order to fully understand the nature of their biological activities. Future research should also focus on the pharmacological and toxicological characteristics of the large portion of untested lichen secondary metabolites, ideally of those compounds that present more important and promising biological activities. Having this information is crucial before thinking about conducting clinical trials using these compounds, which would allow these lichen secondary metabolites to become novel active compounds. Even so, the development of synthetic derivates of these molecules or the use of controlled delivery systems, such as nanoencapsulation, offers another path in the investigation of these metabolites that would reduce the demand of the previous requirements.

Lichens can provide almost endless biologically active compounds, and the species present in Chile are not far behind in this sense. Secondary metabolites that have already been studied in detail have been found and isolated in Antarctic and continental lichens, while several other lesser-known compounds offer the possibility of investigating both their already described biological effects and new discoveries of other biological activities. The information that is already available on these native lichens serves as a first step to updating said knowledge regarding these species and the metabolites that have been isolated from them. In addition, other lichen species present in the Chilean territory could be looked at, thus exploring new findings that encourage the study of the biological activities of their secondary metabolites. Chile is already recognized for its richness in terms of plant species with medicinal properties, and lichens represent an opportunity to discover new species with biological activities and also an opportunity in biochemical and pharmaceutical research at the national level. Recent advancements in biotechnology are allowing laboratory cultures of lichens, opening possible ways to increase the production of bioactive molecules of interest. Thus, lichen species are a key bioresource with high pharmacological potential and may result in novel treatments for current diseases, such as cancer, in the near future.

## Figures and Tables

**Table 1:** Secondary metabolites of pharmaceutical interest found in lichens from Chile.

Secondary Metabolite	Lichen Species
Usnic acid	*Cladonia lepidophora* [25], *Cetraria aculeata* [25,29], *Hypogymnia lugubris* [27], *Ochrolechia antarctica* [25,29], *Ochrolechia frigida* [25], *Parmelia saxatilis* [25], *Protousnea malacea* [23], *Protousnea magellanica* [24], *Psoroma hypnorum* [25,29], *Ramalina terebrata* [25,29], *Rhizoplaca aspidophora* [25,29], *Sphaerophorus globosus* [25,29], *Stereocaulon alpinum* [25,29], *Umbilicaria antarctica* [25].
Atranorin	*Buellia cladocarpiza* [25,29], *Catillaria corymbose* [25,29], *Cladonia cornuta* [25,31], *Cladonia gracilis* [25,31], *Haematomma erythromma* [25,31], *Hypogymnia lugubris* [27], *Lecanora atra* [25,29], *Parmelia saxatilis* [25,29], *Psoroma contextum* [30], *Psoroma hypnorum* [25,29,31], *Psoroma tenue* [25,29,30], *Stereocaulon alpinum* [25], *Umbilicaria antarctica* [25].
Norstictic acid	*Acarospora macrocyclos* [25,31], *Bryoria chalybeiformis* [25,31], *Psoroma* genus (*P. contextum, P. hypnorum, P. tenue*) [25,30], *Rinodina petermanii* [25,31].
Gyrophoric acid	*Ochrolechia frígida* [25], *Ochrolechia deceptionis* [25,29,31], *Placopsis contortuplicata* [25], *Umbilicaria antarctica* [25].
Vicanicin	*Erioderma chilense* [28], *Psoroma* genus (*P. contortum, P. dimorphum, P. leprolomun, P. microphyllizans, P. pallidum, P. pholidotoides, P. pulchrum, P. sphinctrinum, P. soccatum*) [26,30].
Protolichesterinic acid	*Cetraria aculeata* [25,29].
Fumarprotocetraric acid	*Cladonia cornuta* [25,29].
Diffractaic acid	*Protousnea magellanica* [25].
Sphaerophorin	*Sphaerophorus globosus* [25,29].
Psoromic acid	*Rhizocarpon geographicum* [25,31].
Variolaric acid	*Ochrolechia antarctica* [25,29], *Ochrolechia deceptionis* [25,29,31].
Lobaric acid	*Stereocaulon alpinum* [25,29].
Salazinic acid	*Parmelia saxatilis* [25,29].
Physodic acid	*Hypogymnia lugubris* [27].

**Table 2:** Results of half maximal inhibitory concentration (IC_50_) obtained from experimental studies with physodic acid on cancer cell lines.

Cancer Cell Line	Evaluated Time (H)	IC_50_ Results (µg/mL)	References
LS174 (colon carcinoma)	72	17.89	[86]
FemX (human melanoma)	72	19.52	[86]
MCF-7 (breast cancer)	72	34.06	[88]
T47D (breast cancer)	72	35.47	[88]
MDA-MB-231 (breast cancer)	72	44.18	[88]
A-172 (glioblastoma multiforme)	48	61.37	[90]
HeLa (cervical cancer)	72	66	[87]
U-138MG (glioblastoma multiforme)	48	68.36	[90]
T98G (glioblastoma multiforme)	48	72.15	[90]
HepG2 (hepatic cancer)	72	166.15	[89]

**Table 3:** Biological activities of less known lichen secondary metabolites.

Secondary Metabolite	Associated Species	Documented Biological Activities
Divaricatic acid	*Protousnea malacea*, *Lecanora frustulosa*	Cytotoxic activity against melanoma cell lines [94]
Evernic acid	*Evernia prunastri*	Antioxidant and in vitro cytoprotective activity on neuron-like cells against hydrogen peroxide damage, alongside an increase in antioxidant enzymes [45] Cytotoxic activity on melanoma and colon cancer cell lines, with cell cycle arrest on the sub-G1 apoptotic phase [86] Moderate inhibition of mPGES-1 [93]
Lecanoric acid	*Umbilicaria antarctica, Ochrolechia androgyna*	Antioxidant activity of superoxide anion scavenging [66]
Norstictic acid	*Toninia candida*, *Xanthoparmelia chlorochroa*, *Parmotrema, Pseudoparmelia,* and *Usnea* spp.	Antioxidant activity on DPPH and superoxide radicals [111] Cytotoxic activity on melanoma and colon cancer cell lines [111] Anti-proliferative activity on Burkitt’s lymphoma cell lines with phase G0/G1 cell cycle arrest and p53 expression increase [81]
Olivetoric acid	*Pseudevernia furfuracea*	Cytotoxic activity against glioblastoma multiforme cell lines with oxidative damage towards DNA [112] Antioxidant activity in human fibroblast cell cultures [92] Inhibitory activity of mPGES-1 [93]
Pannarin	*Sphaerophorus globosus*, *Psoroma* genus	Protective activity of plasmid DNA pBR322 against UV radiation and free radicals [113] Anti-proliferative activity against melanoma cell lines with DNA fragmentation, caspase-3 activation, and intracellular increase in ROS [113]
Perlatolic acid	*Cladonia portentosa*	Cytotoxic activity against melanoma cell lines [94] Inhibitory activity of mPGES-1 [93]
Psoromic acid	*Alectoria, Psoroma,* and *Usnea* spp.	Anti-proliferative activity against melanoma and glioblastoma multiforme cell lines [94,112] Concentration-dependent antioxidant activity towards free radicals [43] Inhibitory activity of HMGR (non-competitive inhibition) and ACE (mixed inhibition) [43]
Rhizocarpic acid	*Rhizocarpon geographicum*	Inhibitory activity against AGEs [70]
Sekikaic acid	*Protousnea malacea*	Antioxidant activity with superoxide anion scavenging [66] Inhibitory activity of α-glucosidase (competitive inhibition) and β-glucosidase (non-competitive inhibition) [52]
Sphaerophorin	*Sphaerophorus globosus*.	Protective activity of plasmid DNA pBR322 against UV radiation and free radicals [113] Anti-proliferative activity against melanoma cell lines with DNA fragmentation, caspase-3 activation, and intracellular increase in ROS [113]
Variolaric acid	*Ochrolechia* spp.	Possible photoprotective booster against UVA radiation [59] Inhibitory activity against AGEs [70]
Vicanicin	*Psoroma* spp.	Anti-proliferative activity against cervical cancer cell lines (IC_50_ of 67 µM) [32] Concentration-dependent anti-proliferative activity against prostate cancer cell lines with caspase-3 activity increase, and modulation of proteins associated with apoptosis [80]
Vulpinic acid	*Vulpicida pinastri*	Antioxidant activity, DPPH, and superoxide scavenger, as well as having UVA filter properties [47] Photoprotective activity against UVB radiation in keratinocytes cell lines [114] Protective activity against ROS (15 µM) on endothelial cell lines under treatment with hydrogen peroxide [115] Anti-proliferative activity on breast, colorectal, rhabdomyosarcoma, and hepatocellular cancer cell lines, with modulation of proteins associated with apoptosis [116,117] Anti-proliferative activity against prostate cancer cell lines with phase G0/G1 cell cycle arrest, caspases activation and modulation of proteins associated with apoptosis [118]

## Data Availability

The data that support the findings of this study are openly available in Pubmed, ScienceDirect, and SciELO. The authors confirm that the data supporting the findings of this study are available within the respective article.
